# Early Medieval Muslim Graves in France: First Archaeological, Anthropological and Palaeogenomic Evidence

**DOI:** 10.1371/journal.pone.0148583

**Published:** 2016-02-24

**Authors:** Yves Gleize, Fanny Mendisco, Marie-Hélène Pemonge, Christophe Hubert, Alexis Groppi, Bertrand Houix, Marie-France Deguilloux, Jean-Yves Breuil

**Affiliations:** 1 French National Institute for Preventive Archaeological Research (INRAP), Bron, France; 2 University of Bordeaux, UMR 5199 PACEA, Equipe Anthropologie des Populations Passées et Présentes, Allée Geoffroy ST Hilaire, Pessac Cedex, France; 3 University of Bordeaux, Plateforme Génome Transcriptome, Centre de Génomique Fonctionnelle de Bordeaux, Bordeaux Cedex, France; 4 University of Bordeaux, Bordeaux Bioinformatics Center (CBiB), Bordeaux Cedex, France; 5 French National Institute for Preventive Archaeological Research (INRAP), Nîmes, France; 6 UMR 5140, Archéologie des Sociétés Méditerranéennes, Lattes, France; Museo Nazionale Preistorico Etnografico 'L. Pigorini', ITALY

## Abstract

The rapid Arab-Islamic conquest during the early Middle Ages led to major political and cultural changes in the Mediterranean world. Although the early medieval Muslim presence in the Iberian Peninsula is now well documented, based in the evaluation of archeological and historical sources, the Muslim expansion in the area north of the Pyrenees has only been documented so far through textual sources or rare archaeological data. Our study provides the first archaeo-anthropological testimony of the Muslim establishment in South of France through the multidisciplinary analysis of three graves excavated at Nimes. First, we argue in favor of burials that followed Islamic rites and then note the presence of a community practicing Muslim traditions in Nimes. Second, the radiometric dates obtained from all three human skeletons (between the 7th and the 9th centuries AD) echo historical sources documenting an early Muslim presence in southern Gaul (i.e., the first half of 8th century AD). Finally, palaeogenomic analyses conducted on the human remains provide arguments in favor of a North African ancestry of the three individuals, at least considering the paternal lineages. Given all of these data, we propose that the skeletons from the Nimes burials belonged to Berbers integrated into the Umayyad army during the Arab expansion in North Africa. Our discovery not only discusses the first anthropological and genetic data concerning the Muslim occupation of the Visigothic territory of Septimania but also highlights the complexity of the relationship between the two communities during this period.

## Introduction

**Fig 1:**
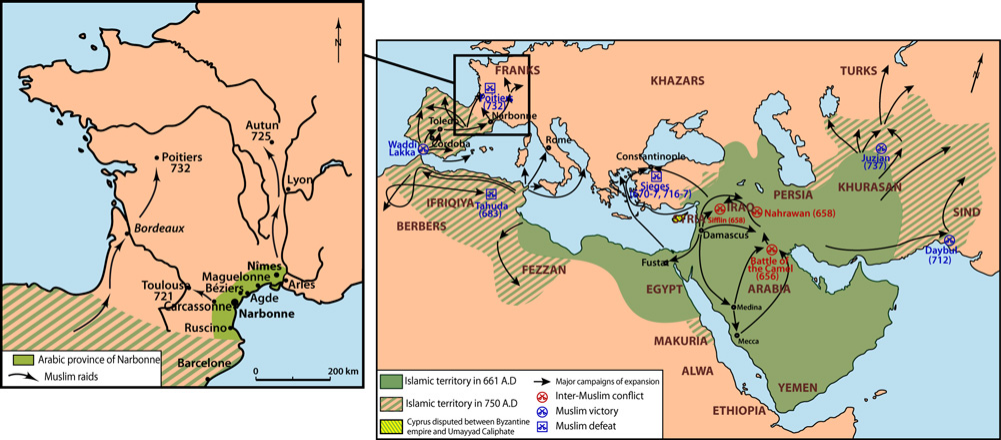
Map of the Arab empire extension and zoom on the Septimania and the north-western Arabic raids. (Infography G. Devilder adapted from [13]).

The rapid expansion of the Arab Empire during the Muslim conquests resulted in the formation of one of the most important empires in world history, extending from the west bank of the river Indus to the shores of the Atlantic Ocean (Fig 1). The Arab-Muslim expansion represented a major politico-religious change during the early Middle Ages in the Mediterranean region. In the western part of the Mediterranean, Arab armies expanded quickly across North Africa and incorporated numerous native Berbers populations, which rapidly adopted the Islamic religion and represented the bulk of Muslim troops who later conquered Southwest Europe [1–4]. The Umayyad army invaded the Iberian Peninsula via North Africa in 711 AD and rapidly conquered the Visigothic kingdom, which, from the 5th to the 8th centuries AD, spread across what are now southwestern France and the Iberian Peninsula. Their arrival led to a cultural transformation and substantially modified relations between Western European societies that were being reorganized after the collapse of the Western Roman Empire. The Muslim occupation of Spain and Portugal is well-documented by abundant written and archeological sources that carefully traced the history of *al-Andalus* between the 8th and the 15th centuries AD [1–2]. In the funerary context, archeological data have highlighted peculiar burial practices that clearly correspond to Muslim practices [5–8]. Interestingly, these specific practices (including, for example, the systematic deposit of bodies on the right-hand side and oriented toward Mecca) demonstrate that Islamic-style graves appear to have persisted from the medieval period to the present day [9–10]. Finally, the medieval population in *al-Andalus* has also been genetically documented through the analysis of human remains originating from three archeological sites in Andalusia and dating to the 12th-13th centuries [11], more than 500 years after the conquest. Although this study doesn’t document the first Muslim occupation, the data provide persuasive evidence of an African maternal contribution in this historic sample (20% of mitochondrial lineages), a contribution that is, interestingly, still seen in the extant populations of the region (at lower frequencies, however; [12]).

In contrast, the Muslim presence beyond the Pyrenees is far less documented, which is likely linked to a brief occupation period (Fig 1). The Umayyad army crossed the eastern Pyrenees in approximately 719 AD. Far from the standard depiction of the famous Battle of Poitiers (or of Tours; 732 AD) that saw Charles Martel lead his Frankish troops to victory over the Umayyad Caliphate army, a far more complex historical account exists beyond what is found through the study of textual sources [14–16]. Notably, certain medieval chronicles (e.g., the chronicle of Moissac) redraw the passage of “Saracens” and highlight the presence of Islamic populations or garrisons in the Visigothic territory of Septimania (in southern France) involving alliances with and protection of the local population, sometimes against a common enemy from the North, i.e., the Franks [13, 17–18]. In 720 AD, Narbonne (under the name of Arbûna) became the seat of a wâli (governor) and was used as a base for razzias. In 759 AD, Pippin the Younger besieged Narbonne, which soon capitulated, and in 760 AD, the Franks took Septimania.

The Islamic presence in Septimania is archeologically documented only by rare ceramics, Arabic coins [19–21] or seals [22]. Nevertheless, these rare materials do not allow for a distinction between trade, travel by Muslim troops or long-term settlements. If written sources indicate that the “Saracens” were able to stay in Septimania for several decades, at this point, we have been ignoring all data regarding the nature of this occupation. The discovery in 2006 of three Muslim burials in Nimes (Languedoc-Roussillon, France) immediately appeared as a unique opportunity to document the “Saracen” settlement in the South of France. A multidisciplinary study was then developed combining (i) archaeological analyses to characterize the site funerary practices, to determine the burials dating, and to discuss the burials' integration in the Nimes funerary context, (ii) anthropological analyses to test the potential attribution of the individuals to Arab army soldiers (through sex and age individuals' characterization, and through the search of potential osteological evidence of combat) and (iii) palaeogenomic analyses to provide biological arguments concerning individuals' origins. The multidisciplinary analyses conducted on these burials offer new data concerning the Muslim occupation in the Visigothic territory of Septimania, unraveling the complex relationship between early medieval western and Arab-Muslim societies.

## Material and Methods

### Excavation

**Fig 2:**
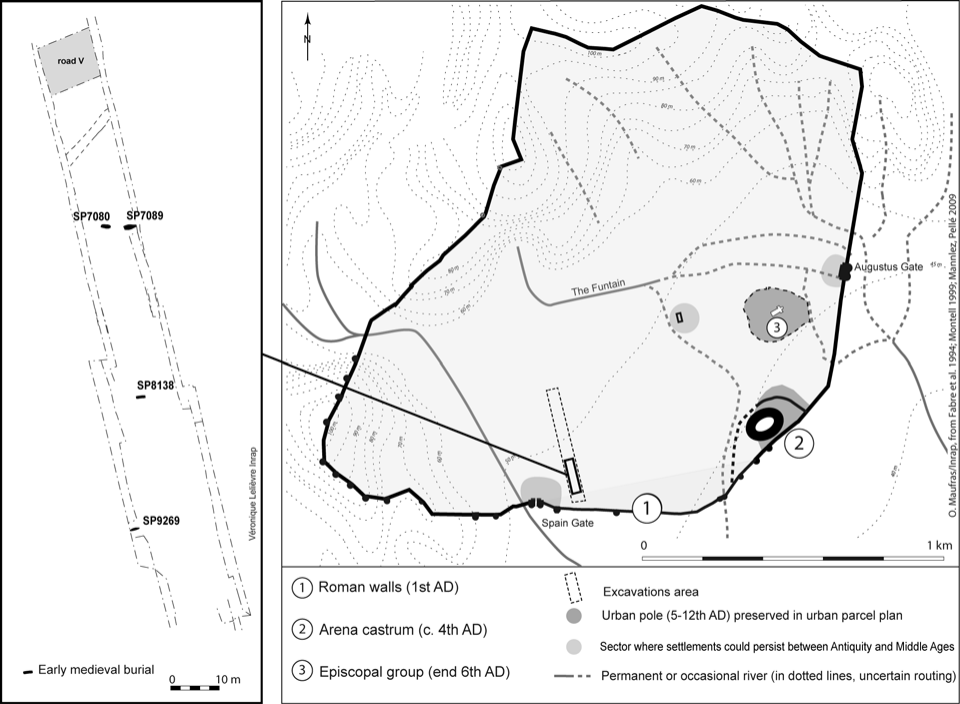
Map of the medieval town of Nimes, with a zoom on the excavations area that revealed the Muslim burials SP7080, SP7089 and SP9269 (analysed in the present study) and the burial SP8138. (Infography G. Devilder from [43]).

In 2006–2007, preventive excavations led by the French National Institute for Preventive Archeology (INRAP) in the western periphery of the medieval town of Nimes (situated on the present-day Avenue Jean-Jaures) revealed about twenty medieval and modern graves scattered across the countryside (Fig 2). This area, which was documented as a Roman quarter of Nimes (with a typical urban landscape) progressively changed into a zone of fallow lands and was home to a mix of cultures after the 3rd century AD. The excavation of the archaeological site, as well as its study was authorized by order of the prefect (N 06/76–6474).

Our attention was particularly attracted by three peculiar graves: SP7080 and SP7089 that are 2.5 meters apart and SP9269 situated 60 meters to the south (Fig 2). Archeothanatological methods [23–24] were used to excavate and study the three different funerary structures (S1 File). Based on the recorded position of bones in the grave, archeothanatology aims to determine the original position of the body, the position of the funerary artifacts, the relative chronology of deposits found in the grave and the architecture of the burial when it first took place. The dislocation and displacement of the bones allow experts to deduce whether the body was directly covered with earth or protected in an empty space, such as with a coffin or cover.

### Anthropological analyses

For the sex attribution, we applied two recent and reliable methods based on hip bone: a morphological approach method [25] and the DSP (i.e., Probabilistic Sex Diagnosis in French, [26]). The individual’s age at death was estimated via sacropelvic surface observation according to the Schmitt method [27], and stature estimation was carried out from long bones using Cleuvenot and Houët [28] formulae. The skeletons US7083 (burial SP7080), US7160 (burial SP7089) and US9270 (burial SP9269) are stored in the laboratory of biological anthropology (UMR 5199 PACEA) of the University of Bordeaux (Gironde department, France).

### Palaeogenetic/Palaeogenomic analyses

All DNA extraction and library preparation was performed in the DNA facilities of the laboratory of Past and Present Populations Anthropology (University of Bordeaux, UMR PACEA) (S2 File) using standard precautions to minimize the risk of exogenous DNA contamination.

DNA was extracted from one tooth collected in situ from each of the three individuals. Each sample was ground, and 200–400 mg of the resultant powder was used for DNA extraction according to the procedure of Mendisco *et al*. [29] using a NucleoSpin® Extract II kit (Macherey-Nagel, Düren, Germany). Three independent DNA extractions were carried out for each sample. Classical palaeogenetic analyses implied the sequencing of a 393-bp fragment of the mtDNA HVR-1 (through the amplification of four short overlapping fragments) and the genotyping of 27 mitochondrial and 10 Y chromosome SNPs (Y-SNPs) using the iPLEX technology (Sequenom) (S1 Table) [29]. All protocols used in the analysis have been previously described in Mendisco *et al*. [29]. Concerning the palaeogenomic analyses, the complete mitochondrial genome and approximately 450 Y-SNPs were enriched by an in-solution hybridization capture using a SureSelect (Agilent) customized target enrichment protocol. The libraries were produced from 50 μL of DNA extract following the SureSelect (Agilent) protocol. The libraries were sequenced on an Illumina’s MiSeq sequencing system. The methods used are detailed in S2 File.

## Results and Discussion

### Three burials with clear evidence of Muslim funerary customs

**Fig 3:**
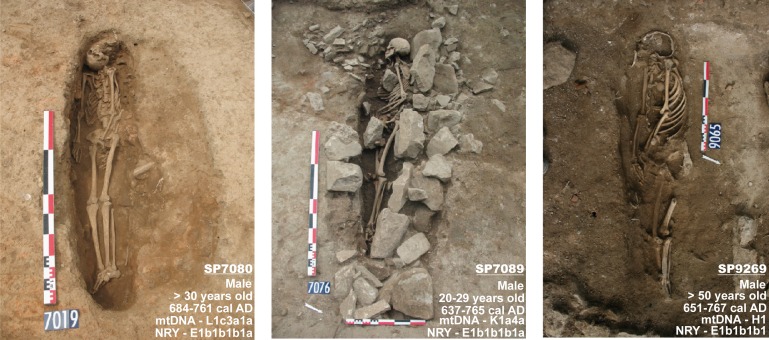
In situ photographs of the Nimes burials, with a synthesis of age and sex of individuals, radiocarbon dates, maternal and paternal lineages. Note that the number near the funerary pit is the recording number of the picture. The stones around the burial SP7089 correspond to a roman wall and some stones were reused to close the funerary pit.

The graves SP7080, SP7089 and SP9269 present a number of common and specific characteristics that were not recorded in other medieval burials in this area. In each of the graves, the body, which may have been wrapped, was directly placed into the pit on its right-hand side facing southeast (in the direction of Mecca). The upper limbs were generally extended, and the lower limbs were extended and sometimes crossed. The burial practices and the position of the bodies clearly correspond to medieval and modern Muslim burial customs [8] (Fig 3).

In at least two cases (SP7080 and SP7089), the burial pit was dug with a lateral niche closed off by slabs or stones (S1 Fig). In Muslim burial traditions, this shape corresponds to the typical *al-lahd* burial as opposed to *al-shaqq* burials (a single trench) [7–8]. Burials with identical shapes were recorded in the early Middle Ages in the northwestern Mediterranean area—i.e., Spain (e.g., [6]), Portugal [30] and Sicily [31]—and they have been systematically interpreted as Islamic graves. The funerary practices observed in Nimes, in particular for the position of the body, are very close to those observed in necropolises dated from the Conquest in the Iberian Peninsula [32–34]. We nevertheless note that if *al-lahd* burials become more widespread during later periods [6], they are not encountered in all Muslim cemeteries contemporaneous to Nimes site. For example, the documentation from the site of Plaza del Castillo, a large early medieval Islamic cemetery (8th c. A.D.) in Pamplona, rather indicates *al-shaqq* burials closed by laying down flat slabs [32].

Interestingly, several observations suggest that the Muslim graves were not isolated or excluded from general funerary space. First, although the three Muslim graves were discovered in an area surrounding the city (outside the borders of the early medieval town), they were found in a distinct rural area situated inside a Roman enclosure (demarcated by stone walls) and between urban poles (Fig 2). Because the Roman walls were still partially visible in the early Middle Ages, we can speculate that this funerary zone was in some way still linked to the city. Moreover, the Muslim graves were not isolated in the area because other early medieval graves were found in the suburb of Nimes, corresponding to a well-known phenomenon in the early Middle Ages [35]. We also note that graves SP7080 and SP7083 were situated 27 meters south of a medieval access road to Nimes. Finally, we note the possible presence of a Christian grave (SP8138, dated between the 8th and 9th centuries AD, containing a body buried on its back with the head facing west) between the two groups of Muslim graves (Fig 2).

### The earliest medieval Muslim graves known in France

Five human bone fragments from the three graves underwent direct radiocarbon dating (S2 Table). The dates obtained, confirmed by two dating labs, cluster tightly and range between the 7th and the 8th centuries AD. These dates suggest that the remains are the earliest medieval Muslim graves known in France, considering the few other Islamic graves reported thus far in southeastern France were dated from the 13th century AD (in Marseille; [36]) and possibly from the 12th century AD (in Montpellier; [37–38]).

### Muslim presence confirmed by textual sources

Textual sources, specifically the Moissac and Uzès chronicles, offer a significant testimony to the complex and unstable historical context of the Nimes region during the early Middle Ages. They notably attest to a Muslim presence or travel in Nimes between 719 and 752 AD. The city—at that time called Niwmshû or Namûshû by Muslim authors—would have initially been taken by the “Saracens,” possibly at the end of 719, but was rapidly retaken by Eudes, Duke of Aquitaine, in 721. In 724 or 725, the inhabitants of Nimes surrendered, offering little resistance to Ambissa, or Anbasa b. Suhaym al-Kalbi, the new governor of Spain [17, 39–40]. Despite the city’s devastation by Charles Martel in 737, Nimes’ Muslim presence may have persisted after this date. Finally, in 752, a local Goth leader named Ansemundus (or Misemundus) delivered four cities, including Nimes, to Pepin the Short, marking the start of the final conquest of Septimania by the Franks.

### Three adult males of North African ancestry

An anthropological analysis shows that the three skeletons are those of male adults (S1 File). Although it is difficult to be certain of the biological identity of these individuals, several anthropological characteristics can be highlighted. The skeletons did not show any marks indicating death resulting from fighting. The skeleton from SP7080 displayed an incomplete fusion between the right pisiform bone and the hamate bone (S2 Fig). This extremely rare fusion, mainly seen in African populations, suggests an African origin for the Nimes human remains (e.g., [41–42]). Nevertheless, no dental decoration, potentially testifying a North African origin and already described on a skeleton discovered in the site of Plaza del Castillo in Pamplona [43], could be observed on the Nimes individuals.

**Fig 4:**
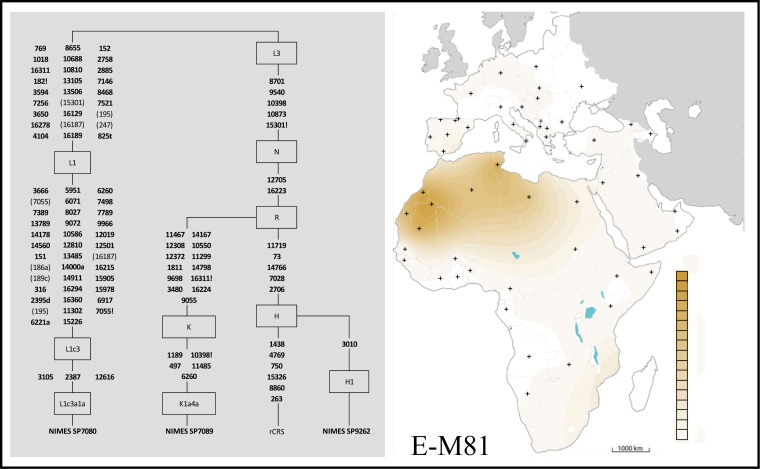
Simplified phylogeny of the mitochondrial lineages (L1c3a1a, K1a4a and H1) and geographic repartition of the Y-chromosome lineage E-M81 characterised on the SP7080, SP7089 and SP9269 human remains.

Paleogenetic and palaeogenomic analyses were conducted on the three Nimes individuals to better understand their bio-geographical origin. To date, only one publication has described the mitochondrial lineage of medieval human remains originating from archeological sites in *al-Andalus* [11]. These samples date from the 12th-13th centuries AD and, as such, provide a snapshot of the local population gene pool several centuries after the establishment of Muslim domination over the Iberian Peninsula. Thus, the genetic analysis of the Nimes human remains provided a unique opportunity to identify the genetic lineage carried by the individuals associated with the initial part of the Muslim conquest in Western Europe. Using a specific capture of mitochondrial genomes and more than 450 Y chromosome SNPs (Y-SNPs; see S2 File for analyses details), we managed to characterize the complete mitogenomes from all three individuals as well as partial Y-SNPs profiles (S3 Fig, and S3 Table). These results were completely consistent with the classical analyses initially conducted on the human remains (mtDNA and Y-chromosome SNPs analyses, and sequencing of HVR-1; S4 Table) and identified three distinct mtDNA haplotypes: L1c3a for SP7080, which is typically found in African populations; K1a4a for SP7089 and H1 for SP9262, which are more widely distributed across different regions in Europe and Asia but also occur in Africa (Fig 4). The current distribution of these mitochondrial haplotypes is presented as supporting information (S4 Fig). Even if the capture and enrichment of Y-SNPs was less effective, they indicated the presence of the same typical North African haplotype E1b1b1b-M81 [12, 44] in all three males’ DNA samples (S3 Table). It is worth noting that the E-M81 lineage is particularly well-represented among the North African Berber communities, with frequencies up to 70% [45–46] (Fig 4). The significant presence of this haplogroup outside North Africa—i.e., in extant populations of Iberia, Italy and Sicily (S4 Fig)—relates directly to the long-term Arab rule in these regions [46]. If the paternal lineage E-M81 and the maternal lineage L1c3 characterized implies with a high degree of probability a North African origin for all Nimes individuals, we have to note that the large distribution of mtDNA lineages H1 and K (both in North Africa and Europe) do not permit to drive any clear conclusion concerning individuals' maternal ancestry. Indeed, the determination of these maternal lineages on Nimes burials may be both the result of a direct North African maternal origin and the result of admixture between migrating Muslims and local European women. If the low discriminatory power of mtDNA does not permit us to decide between both hypotheses, genome-wide data may permit to precise individuals' ancestries in the next future. Nevertheless, if admixture between Muslims and European women is well established for later *al-Andalus* periods (genetically established for sites in Andalusia dating to the 12^th^-13^th^ centuries; [11]), such admixture had not been raised so far for the very first Muslim groups arriving in Europe. If admixture with local women was confirmed concerning Nimes individuals, these data would constitute the most ancient evidence of admixture in the *al-Andalus* context.

Mutations are transitions unless specified. Transversions are indicated by an A, C, G, T after the nucleotide position, and mutations back to the CRS nucleotide are indicated by a "!". Note that the positions that have 4-fold and more coverage are indicated in bold and the positions that have less than 4-fold coverage are noted in brackets.

### Synthesis of multidisciplinary study and historical perspectives

Given the multidisciplinary nature of this study, which combines archeological, anthropological, historical and palaeogenomic discussions, we attribute the three Muslim graves from the site of Nimes to individuals with paternal ancestry from the Maghreb. These burials, dated between the 7th and the 8th century AD, represent the earliest medieval Muslim graves known in France (Fig 3). This discovery has had a resonance, especially in available textual sources that indicate a few decades of Muslim presence in Nimes, between 720 and 752 AD. We suggest that the graves discussed in this study can provide further insight into the nature of this Muslim presence. Indeed, the discovery of funerary rites faithful to Muslim customs offers evidence indicating the presence of a community that was familiar with and practiced Muslim customs in Nimes during this period.

Because the palaeogenomic data support a North African paternal ancestry of the three individuals from the graves, we believe that they were Berbers integrated into the Arab army during its rapid expansion through North Africa. Such conclusions are in perfect accordance with the ones deriving from the isotopic analyses conducted on two individuals from Plaza del Castillo in Pamplona [47]. Because the remains may be those of soldiers, it is worth noting that the bodies deposited in the graves were carefully buried (with clear respect for funerary customs) and did not present any osteological evidence of combat (which do not testify to deaths resulting from combat), as already pointed out for Islamic necropolises in Spain [48]. Moreover, in the cemetery of Plaza del Castillo in Pamplona (dating from the Conquest) adults of both sexes (with notably one female individual showing intentional dental modification testifying of an African origin) and children were discovered, suggesting that family groups or camp followers participated to the early Muslim population [40, 47].

Despite the low number of Muslim graves discovered, we believe that these observations provide strong evidence for either the establishment of a garrison or a more long-term establishment of Muslim communities in Nimes. Moreover, the results we discuss demonstrate that a few years after their integration into the Muslim world, North African populations were interred according to Islamic customs. This observation lends strong support to the quick conversion of the Berber populations and testifies to the velocity of the politico-religious changes involved in the Arab Conquest.

The absence of other archeological testimony of the Islamic presence in Nimes can be easily explained by the brief Muslim occupation. We must nevertheless note that the archeological excavation at Place du Chapitre in the Nimes medieval center [49] resulted in the discovery of a grave in which the body was deposited on its right-hand side and was stratigraphically dated between the end of the 5th century and the 9th century AD. The question of the attribution of this grave to the Muslim occupation remains open. It is also worth noting that this subtle archeological testimony echoes the absence of any noticeable genetic heritage from these Muslim groups in the modern-day French population. The genetic impact of the Muslim occupation on the European gene pool has been assessed by analyzing the extant European gene pool (mainly from Southern Europe). For example, the analysis of extant populations in Iberia has noted the presence of mitochondrial haplogroups of North African origin at low frequencies. Authors have suggested that these lineages may have resulted from the Muslim occupation of the Peninsula but also from a more ancient gene flow that may have occurred during prehistoric times [50–51]. Apart from the mitochondrial haplogroup H1, the maternal and paternal lineages detected in the three Nimes individuals are relatively rare in modern-day France [52]. In comparison to the Iberian Peninsula or Italy, it appears clear that the genetic impact of the Arab rule was less significant in France.

Finally, several observations suggest that Muslim graves were not excluded from the funerary space or isolated. Thus, if the three Muslim graves of Nimes were not found in a cemetery, it is not necessarily a sign of exclusion from the community. During the early Middle Ages, the concept of Christian cemetery (understood as the cemetery for all Christians) was built progressively. All graves of Christians were not placed in a holy ground near a church and could have been scattered [35]. Additionally, several historians have proposed that the local populations in Narbonne (certainly in the region) could have accepted a type of protection and may have been allowed to preserve their laws and traditions under Muslim domination [17, 53–54]. If the funerary discoveries at Nimes do not offer answers to these questions, they support the complexity of the relationship between communities during this period, which cannot be summarized in a simple opposition between Christians and Muslims.

### Conclusion

Using a multidisciplinary approach that combines history, archeology, anthropology and palaeogenomics, we discuss the first early medieval Muslim graves discovered in an area north of the Pyrenees. Although a Muslim presence in Septimania was already known through textual evidence, the complete analysis of the graves provides new data concerning the first groups of Muslims that arrived in France. Notably, the analyses confirm the Berber origin of some of the first Muslim troops spreading through Europe and also indicate the co-existence of communities in Nimes practicing Christian and Muslim funerary customs without any clear partition of their respective funerary spaces. These results clearly highlight the complexity of the relationship between communities during this period, far from the cliché depiction still found in some history books.

## Supporting Information

S1 Fig Slabs closing the niche of grave SP7080. Note that the number is the recording number of the picture.
(TIF)

S2 Fig Incomplete fusion between the right pisiform bone and the hamate bone (SP7080). Palmar view (right pisiform bone and the hamate bone), proximal view (right hamate bone) and distal view (right pisiform bone).
(TIF)

S3 Fig Simplified phylogeny of mitogenomes sequenced in this study. Mutations are transitions unless specified. Transversions are indicated by an A, C, G, T after the nucleotide position, and mutations back to the CRS nucleotide are indicated by a "!". The positions that have 4-fold and more coverage are indicated in bold and the positions that have less than 4-fold coverage are noted in brackets.
(TIF)

S4 Fig Maps displaying the geographical distribution of mtDNA and Y-chromosomal haplogroup frequencies characterized on burials SP7080, SP7089, and SP9262. The frequency patterns were generated using the Kriging method in Surfer 8 program (Golden Software, Inc.). Dots indicate sample locations and the scale bars indicate the haplogroup frequency bins. Given the insufficient level of resolution of some mtDNA analysis, we compiled data for mitochondrial lineages H1, K, and L1c3 (see S6 Table for references of the used modern populations). Note that the scale bars are different for each map.
(TIF)

S5 Fig mtDNA and Y Chromosome damage patterns for the three human remains SP7080, SP7089, and SP9262. On the right, the plots shows the base frequency 5’ and 3’ of the reads (the grey brackets corresponds to the reads). Frequencies are shown for A, G, C, and T for the 10 bases 5′ and 3′ of the reads. On the left, the plots shows the nucleotide misincorporation pattern at the first and last 25 bases of mtDNA fragments (C-to-T misincorporations in red, and G-to-A in blue). Note that Illumina MiSeq reads were generated using libraries amplified with Phusion polymerase, limiting nucleotide misincorporations resulting from cytosine deamination (which explains the non-expected profile of misincorporation observed at the first 25 base pairs of fragments).
(TIF)

S6 Fig Mitochondrial genome coverage for the three human remains SP7080, SP7089, and SP9262. (TIF)

S1 File Archaeological and anthropological analyses. (DOCX)

S2 File Molecular analyses. (DOCX)

S1 Table PCR and SBE primers used SNPs typing (iPLEX technology, sequenom). (PDF)

S2 Table Radioarbon dating. (PDF)

S3 Table Mutated Y-SNPs detected for the three human remains analyzed. (PDF)

S4 Table Consensus HVR-1 sequences and SNP retrieved for the three human samples. (PDF)

S5 Table HVR-1 sequences, mitochondrial and Y chromosome SNPs of the researchers involved in this study. (PDF)

S6 Table Details of modern-day populations used for comparison. (PDF)
