# Supplementary figures and images for: Early Medieval Muslim Graves in France: First Archaeological, Anthropological and Palaeogenomic Evidence
Source: PLoS One. 2016 Feb 24;11(2):e0148583. doi: 10.1371/journal.pone.0148583 (PMC4765927; doi:10.1371/journal.pone.0148583)

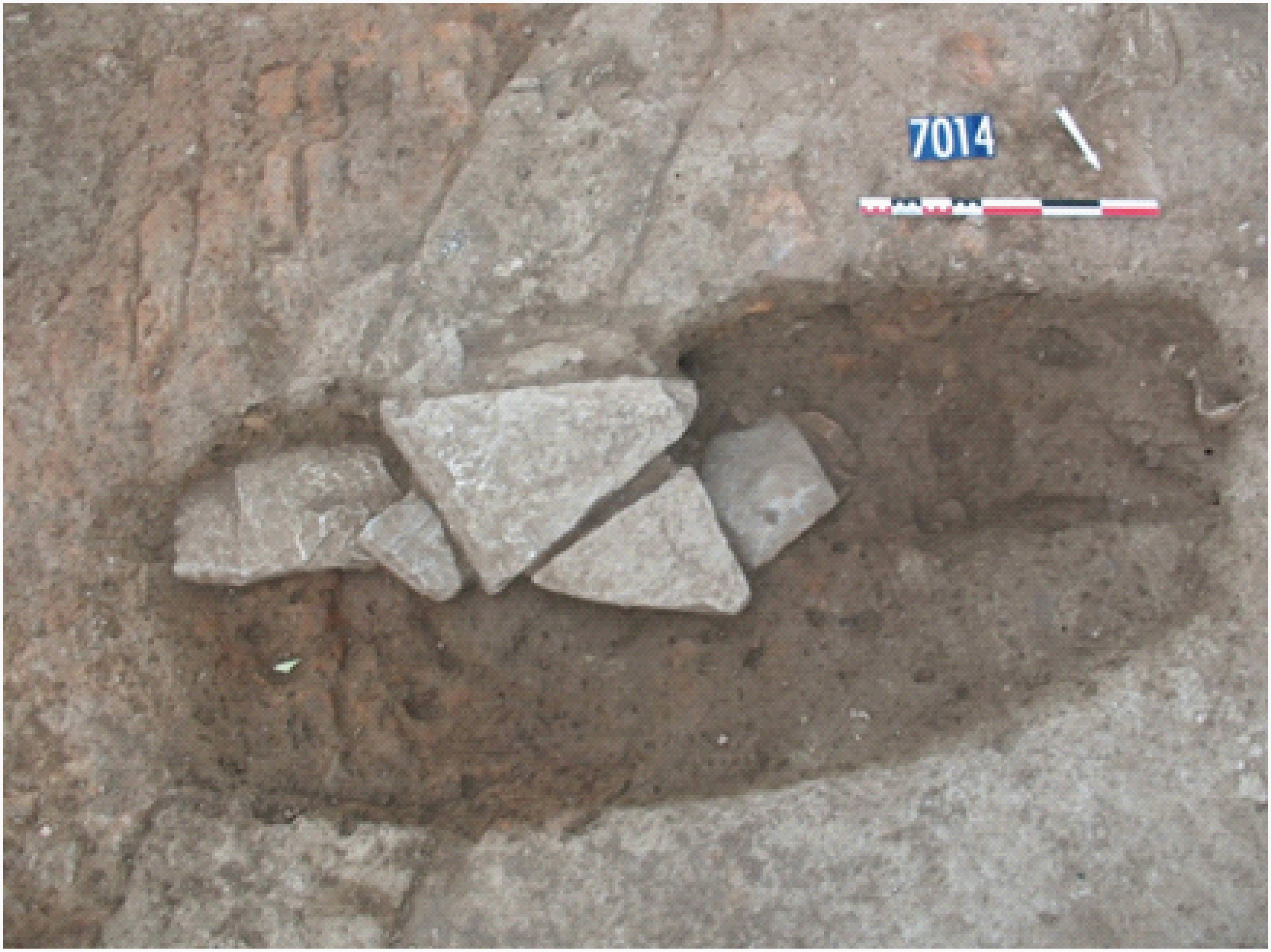

Supplement: S1 Fig — Note that the number is the recording number of the picture. (TIF) [file pone.0148583.s001.tif]

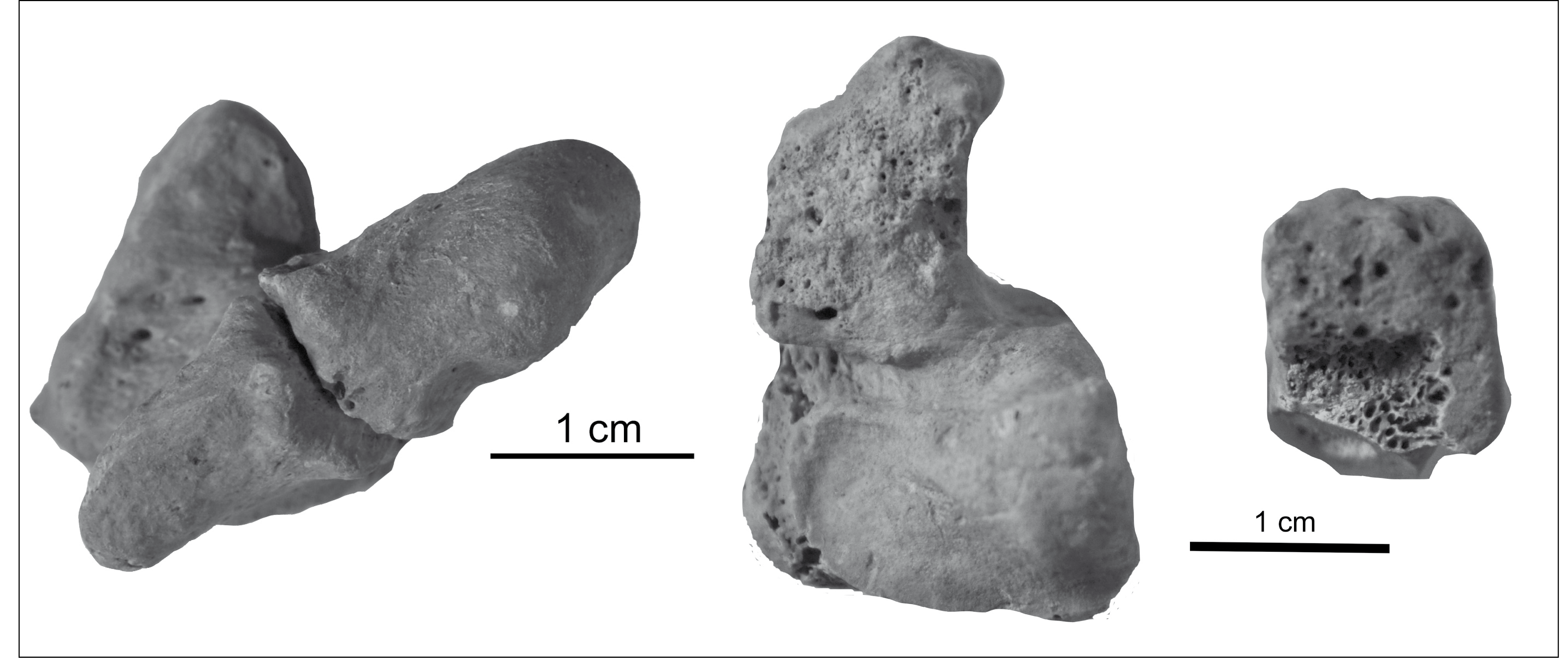

Supplement: S2 Fig — Palmar view (right pisiform bone and the hamate bone), proximal view (right hamate bone) and distal view (right pisiform bone). (TIF) [file pone.0148583.s002.tif]

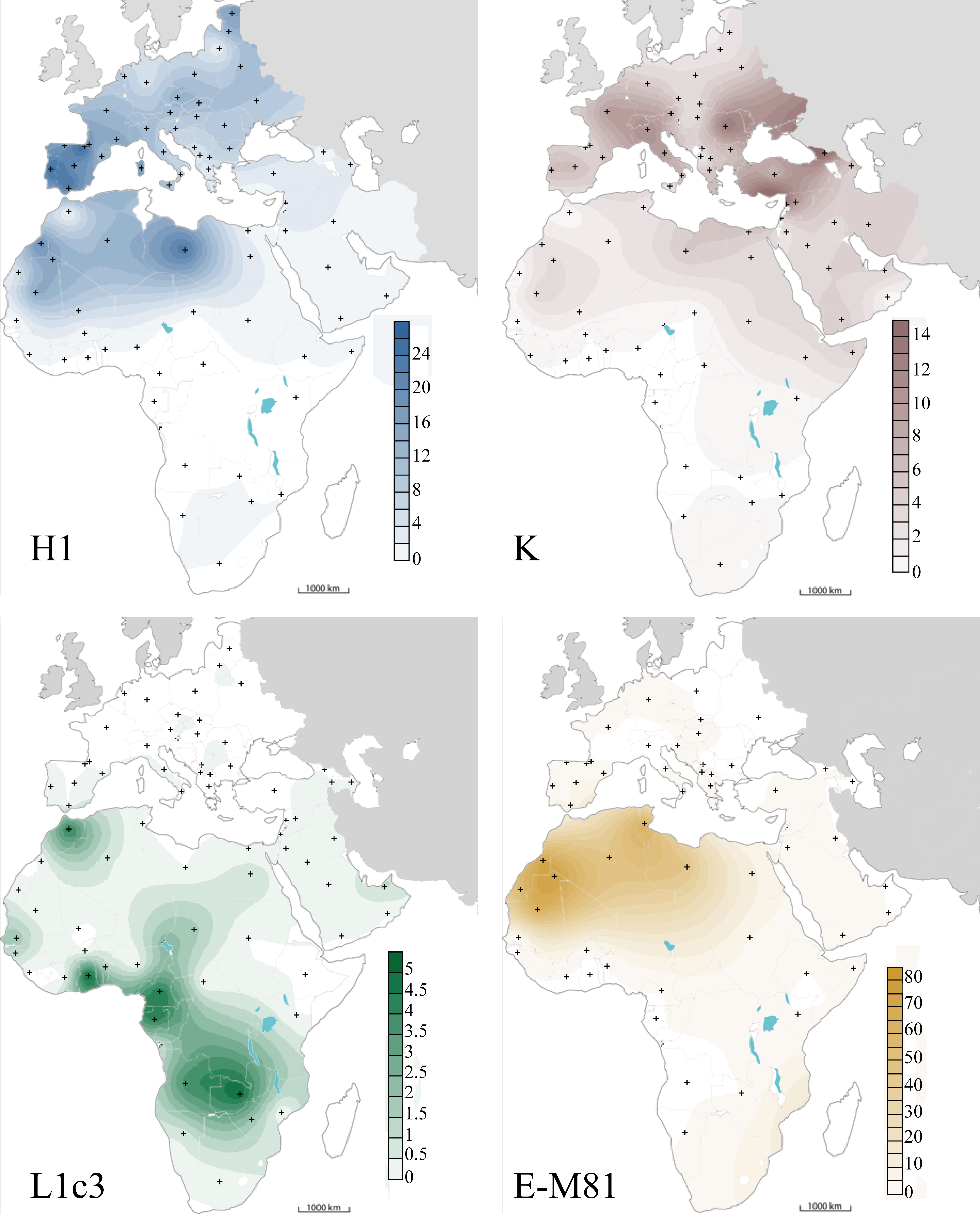

Supplement: S4 Fig — The frequency patterns were generated using the Kriging method in Surfer 8 program (Golden Software, Inc.). Dots indicate sample locations and the scale bars indicate the haplogroup frequency bins. Given the insufficient level of resolution of some mtDNA analysis, we compiled data for mitochondrial lineages H1, K, and L1c3 (see S6 Table for references of the used modern populations). Note that the scale bars are different for each map. (TIF) [file pone.0148583.s004.tif]
