# Supplementary material for: Early Medieval Muslim Graves in France: First Archaeological, Anthropological and Palaeogenomic Evidence
Source: PLoS One. 2016 Feb 24;11(2):e0148583. doi: 10.1371/journal.pone.0148583 (PMC4765927; doi:10.1371/journal.pone.0148583)
Supplement: S1 File — (DOCX) [file pone.0148583.s007.docx]

**Supporting Information File S1**

**Archaeological and Anthropological analyses**

**Archaeo-thanatological analyses**

The three sepulchral pits SP7080, SP7089 and SP9269 present an oblong shape and are orientated northeast / southwest. The graves SP7080 and SP7089 present a niche 0.40 meters wide on their southern part, where the body was placed. Given soil level, it is possible to suspect a shaft pit with a lateral niche. The levelling of the burial SP9269 does not provide any evidence of niche, but the pit narrowness (0.35 meters wide) could get closer to the niche of the two other graves. Thus, it is not excessive to suppose that this burial would present the same shape. In the graves SP7080 and SP7089, the niche was closed by slabs or stones (S1 Fig). The indications of body decomposition in empty space in the graves (e.g., movement of feet bones in SP7089) permit to demonstrate that no sediment was directly deposited on the body.

In all three burials, the body was buried on the right side with the face directed towards southeast. In the grave SP7080, the high position and the dislocation of the cranial skeleton permit to highlight a disturbance of the skull. The preservation of the first cervical vertebra (atlas) in anatomical position testifies of this disturbance after decay of the body [1]. This hypothesis is moreover confirmed by the absence of cover elements in this part of the grave. As a consequence, the skull is not in its original position and, according to the position of the body, we can legitimately propose that the face was in the direction of the southeast. Upper limbs appeared generally in extension with hands in front of the pubis and lower limbs also in extension with the left foot on the right foot.

Due to pits narrowness, it is difficult to detect evidence of shroud or wrapping. Nevertheless, in the grave SP7089 the patella appeared in unstable position and distant from pit walls, what could validate the existence of a sewn shroud or at least wrapping. It is however necessary to remind difficulties linked to the identification of textile wrapping in absence of pins.

**Sex/age determination**

Anthropological analyses permitted to demonstrate that the three skeletons corresponded to male adults. SP7080 skeleton was aged more than 30 years old, SP7089 skeleton was aged between 20 and 29 years old and SP929 skeleton was aged more than 50 years old.

The stature was estimated with maximum length of femur to 169,2+/- 4 cm for SP7080, to 184,5 +/- 4 cm for SP7089 and to 166,3 +/- 4 cm for SP9269.

**Paleopathological evidences**

No caries were observed although the individuals correspond to three adults, with even one old adult. Few osseous pathologies were observed. The left upper limb of the skeleton SP9269 presents several traumas. First, the median part of his clavicle diaphysis was broken and the absence of fracture reduction was responsible for the appearance of a pseudoarthrosis. Second, the distal part of his ulna diaphysis reveals the result of a healed fracture. Both traumas may very well be the result of a single event like a fall during the life of this individual.

It is worth noting that two of the skeletons (SP9269, SP7089) have a lumbarization of the sacred vertebras. A far most particular anthropological character discovered is an incomplete fusion between the right pisiform bone and the hamate bone in SP7080 (S2 Fig). This very rare coalition was described on several occasions in the anthropological literature but was mainly observed in African population.

**Radiocarbon dating**

Five human bone fragments from the three graves were submitted to radiocarbon dating. Two samples were prepared at the AMS C14-Labor Erlangen and three samples at Beta Analytic Miami. Raw dates are corrected for isotopic fractionation and calibrated at 1 and 2σ (standard error) using Intcal13 [2]. The details concerning the dates obtained can be found in S2 Table.

**REFERENCES**

1. Duday H. The Archaeology of the Dead: Lectures in Archaeothanatology. Oxford: Oxbow Books; 2009.
2. Reimer PJ, Bard E, Bayliss A, Beck JW, Blackwell PG, Ramsey CB, et al. IntCal13 and Marine13 Radiocarbon Age Calibration Curves 0-50,000 Years cal BP. Radiocarbon. 2013; 55(4): 1869–1887. DOI: 10.2458/azu_js_rc.55.16947.
