# Supplementary material for: Early Medieval Muslim Graves in France: First Archaeological, Anthropological and Palaeogenomic Evidence
Source: PLoS One. 2016 Feb 24;11(2):e0148583. doi: 10.1371/journal.pone.0148583 (PMC4765927; doi:10.1371/journal.pone.0148583)
Supplement: S2 File — (DOCX) [file pone.0148583.s008.docx]

**Supporting Information File S2**

**Molecular analyses**

**Sample processing.**

All DNA extraction and libraries preparation were conducted in the clean room facilities at the University of Bordeaux (University of Bordeaux, UMR PACEA, Palaeogenetic Platform). All pre-PCR steps were conducted under sterile conditions (high-pressure system, filtered incoming air, UV light irradiation, surfaces cleaned with bleach, and a laminar flow hood), using DNA-free reagents. Workers were required to wear clean-room overalls, shoe protection, facemasks, and gloves. DNA extraction and manipulation (first step), preparation of the PCR reagents (second step), and mixing of the PCR reagents and DNA extracts (third step) were conducted in separate dedicated rooms, which were spatially distant from the post-PCR laboratory where the PCR amplifications and subsequent analyses took place.

Teeth were isolated from mandible in a sterile laminar flow hood, scraped off, cleaned with bleach and finally exposed to UV radiation for 20 minutes on each side.

**DNA extraction.**

DNA was extracted according to a previously described protocol [1]. Briefly, the samples (teeth still included in the jaw) were collected in situ and were submitted to a treatment of bleach and UV radiation. Each sample was ground, and the resultant powder was incubated overnight at 55°C with agitation in a lysis solution (0.5 M EDTA, NaOH, 1-2 mg/mL Proteinase K, and 0.5% N-lauryl sarcosyl). Then, the supernatant was recovered through a silica-based extraction method using the 'NucleoSpin® Extract II kit' (Macherey-Nagel, Düren, Germany). For each sample, three different extracts were obtained.

**SNPs analyses.**

We performed two multiplex SNP typing designed with MassArray assay design software (version 4.0). A total of 27 mitochondrial SNPs and 10 Y chromosome SNPs were targeted through these two multiplex, permitting the characterization of common worldwide as well as European maternal and paternal lineages. We provide a synthetic table of all used primers in S1 Table. We used iPLEX^TM^ Gold technology (Sequenom) protocol, a MALDI-TOF MS-based SNP genotyping technique, to perform genotyping reactions as described in Mendisco et al. [1]. Consensus SNP typing from the coding region of mitochondrial DNA and from the Y-chromosome was deduced from four replicates obtained from two different extracts.

**HVR-1 analysis.**

We amplified four overlapping fragments of the mtDNA HVR-1 control region. These fragments range from 107 to 149 bp, and were amplified with the following primer pairs: L15,989 and H16,158, L16,112 and H16,239, L16,190 and H16,322, and L16,268 and H16,401 [2]. This combination of primers yields a 392 bp long fragment of the HVR-1 region (nps 16,009-16,400). All mutations were established according to the revised Cambridge Reference Sequence (rCRS) [3-4]. The consensus HVR-1 sequences were always deduced from multiple sequences originated from different extracts and PCRs. PCR ampliﬁcations were performed in a 25 µL reaction volume containing 6.5 µM MgCl 2, 0.4 µM dNTP, 0.66 mg/mL BSA, 1 µM each primer, 2.5 µL GeneAmp 10x PCR Buffer (Perkin-Elmer), 0.25 µL DNA extract, and 1.25 U AmpliTaq Gold^TM^. PCR were run for 40 cycles at 94°C for 45 s, 56°C for 45 s, and 72°C for 45 s. PCR products were sequenced with Sanger techniques.

**Library preparation, capture and sequencing.**

Extracts providing most complete SNPs profiles and then presenting the better DNA conservation were selected for NGS analyses. We used Agilent’s SureSelect XT Target Enrichment System for Illumina Paired-End Sequencing Library (Agilent Technologies, USA) on the three ancient samples. The in-solution hybridization capture was customized using information available on the phylogeny of paternal genetic lineages (www.isogg.org/tree/) [5]. Complete mitochondrial genome and Y-chromosomal fragments, including more than 450 single nucleotide polymorphisms diagnostic of all paternal lineages, were targeted in 120 bp fragments and 5x tiling density. Libraries were prepared from 50 μl DNA extract selected from each individual following the SureSelect XT Target Enrichment protocol. Each amplified library was subjected to two enrichment PCRs to obtain enough material for the capture. The captures were conducted following the SureSelect XT Target Enrichment protocol. Sequencing was performed on a single lane of a flow cell on the Illumina’s MiSeq platform.

**Bioinformatic sequence analysis**.

NGS sequences have been first analyzed and filtered through the script IlluQC_PRLL.pl from NGS QC ToolKit (v.2.2.3) [6] with in input the forward and reverse fastq files, the primer/adaptor sequences and the following parameters: cut-off value for percentage of read length that should be of given quality: 70; cut-off value for PHRED quality score for high-quality filtering: 20; keep only paired reads; discard adaptor contaminated reads. The resulting reads were then interleaved with the script interleave-reads from khmer 2.0 package [7] and subsequently treated as single reads. The interleaved reads were filtered to retain only reads < 200nt by using prinseq lite 0.20.4 [8]. The size-filtered reads were mapped on the reference genome (Mitochondrion complete genome NCBI Reference Sequence NC 012920.1 or Homo sapiens chromosome Y GRCh37 p10 Primary Assembly NC 000024.9.fasta) by using BWA 0.7.12 with seed disabled [9]. Sam files were converted to bam file then sorted and indexed by using samtools 1.2 [10]. The script MarkDuplicates from Picard-tools 1.119 was applied to remove duplicates. Quality control of alignments sequencing data was obtained by using Qualimap v2.1.2 [11]. The subsequent chromosome Y and Mitochondrion alignments were analyzed by MapDamage2 [12] with the –reverse option to circumvent the artifact due to the use of the Phusion polymerase during the PCR step (see also S3 Fig). We used contamMix 1.0-10 kindly provided Dr Philip Johnson according to the protocol described by Fu *et al.*, [13]. This script allowed us to estimate the level of exogenous human DNA contamination in the mtDNA sequences. This method compares for each individual the mapping affinities of its mtDNA sequences to its own consensus mitogenome sequence, relative to the mapping affinity of its mtDNA sequences to a dataset of potential contaminants represented by 311 mitogenomes from worldwide populations described in Krause et al, 2010 [14] and available at <https://raw.githubusercontent.com/mpieva/mapping-iterative-assembler/master/misc/mt311.fa>. The mitogenome consensus sequences were made using the samtools 1.2 mpileup -uf function [10]. The resulting vcf file was indexed and the consensus was generated with bcftools from samtools 1.2. Raw reads are then realigned to this consensus with BWA 0.7.12 [9]. The last input file used by contamMix consisted of an alignment of the 311 whole mitochondrial genomes appended with the consensus sequence by using Muscle 3.8.31 [15]. All variable positions (SNP and Indel) were detected by with samtools 0.1.19 using the mpileup command with the following arguments: “samtools mpileup -t DP -B -d10000000 -u -f” and then converted to vcf file through the following command: “bcftools call -m -O v -o”. Complementary analyses (type and count of each variation, coverage per position, etc.) were computed by custom scripts (unpublished).

**Sequence recovery and authenticity.**

The precautions taken for the collection and processing of samples, the pattern of DNA degradation, the consistency of analyses (HVR-1, SNPs, NGS), and the diversity of genetic lineages obtained (distinct from all manipulators lineages, as presented in S5 Table) are arguments allowing us to be confident about the authenticity of the presented results. S4 Table presents the mitochondrial HVR-1 sequences, SNPs genotyping and haplogroups retrieved from the three samples.

Complete mitochondrial genomes were obtained from burial SP9262 (15.325 total mapped reads after duplicate removal, 100% covered at 143.16-fold mean coverage), burial SP7080 (5.719 total mapped reads after duplicate removal, 100% covered at 51.21-fold mean coverage), and burial SP7089 (3940 total mapped reads after duplicate removal, 100% covered at 35.11-fold mean coverage). As expected for aDNA, the capture and enrichment of Y-SNPs was less effective [11]. Considering the 465 Y-SNPs targeted, 8.2%, 5% and 37.2% were covered at 1-fold mean coverage, for samples SP7080, SP7089 and SP9262 respectively (S3 Table). The overall patterns of DNA degradation, were analyzed by MapDamage2 and followed expectations for degraded DNA [12, 16-17] for (i) mtDNA reads obtained from all burials and (ii) for Y-chromosome reads for burials SP7080 and SP9269 (the number of Y-chromosome reads was too low for SP7089) (S5 Fig).

We finally estimated the levels of mitochondrial DNA contamination through the use of contamMix 1.0–10 [13] that estimates the fraction of mitochondrial DNA sequences that match the consensus more closely than a comparison set of 311 worldwide mitochondrial genomes. The contamination level of the 3 libraries analyzed ranged between 4.7% for SP9269 and 24% for SP7080 (15% for SP7089). Despite these contamination rates revealed not negligible (surely linked to the fact that the samples were not collected with all precautions concerning contaminations), all arguments listed before make us confident in the authenticity of the mtDNA and Y haplotypes characterized (see above).

**Determination of mitochondrial and Y haplotypes.**

Haplotypes determined from palaeogenetic and palaeogenomic data are totally concordant (S3 Fig and S4 Table). The proportion of unique mapped reads obtained for the mitochondrial genome from all three burials (between 3960 and 15325) and the percentage of positions with 4-fold and more coverage (between 99.4% and 99.9%; S6 Fig) allowed detection of variants and clear mitochondrial haplotypes determination (Fig 4).

To study the current distribution of mitochondrial haplotypes, and Y haplogroup determined for the three ancient samples, we compiled data from the literature for modern populations from Africa, Europe and the Middle East (representing about 23.370 HVR-1 sequences between nucleotide positions 16,024 and 16,380) (S6 Table).

Ancient SP7080 individual could be unambiguously assigned to the mitochondrial sub-haplogroup L1c3a1a (S3 Fig). The origin of L1c and its subclades as well as the processes leading to their current distribution are still uncertain [18]. The L1c3 mitochondrial subclade, whose origin is probably related to the Bantu expansion [18], is a relatively rare African lineage reaching highest frequencies in West-central and Southern Africa (S4 Fig). Due to the low level of resolution used in most studies available (mainly based only on HVR-1 sequences) it is difficult to clarify the extant distribution of the specific haplotype retrieved on the human remain originating from the sepulture SP7080. Nevertheless, we could note that this specific mitochondrial lineage has been already described in Egypt [19] and Guinea Bissau [20]. According to the genetic data available in the literature, the lineage L1c3 is extremely rare in Europe, as to our knowledge, it has been observed only in a population from Portugal [21]. The individual SP7089 was identified as belonging to a subclade of mitochondrial haplogroup K1 (K1a4a) (S3 Fig). Again, the level of resolution of mtDNA analyses available in the literature is insufficient to determine the exact distribution of this lineage. Anyway, the K mitochondrial haplogroup is distributed throughout Europe, northern Africa and the Near East (S4 Fig). K1a is the most frequent of the K subclades, and comprises numerous branches, including the K1a4 clade which is found predominantly in Europe and the Near East [22]. In Northern Africa, the K1a lineage reaches noticeable frequencies, between 2 and 5% for Moroccan [23] or Egyptian [24-25] groups for example. The third sample SP9262 which presents a transition substitution at nucleotide position 3010 is assigned to the mitochondrial haplogroup H1 (S3 Fig, and S4 Table). This lineage, distributed mainly in western Europe and northern Africa, reached high frequencies among Iberians, Moroccans or Tunisians but also among Tuaregs from Sahel [26] (S4 Fig). The high frequencies of mitochondrial haplogroup H1 in North Africa are explained by a post-glacial expansion of this lineage from the Iberian Peninsula, in the first half of the Holocene [26]. Currently, a mean frequency of 15% of this lineage is observed in French populations [27].

Despite a low coverage of Y-SNPs, we are confident that the three individuals are associated to the same subclade of the Y haplogroup E-M243 (E1b1b1) (S3 Table). More exactly, two of the individuals (SP7080 and SP7089) present a specific mutation at the marker E-M81 (diagnostic of E1b1b1b1a), while the third (SP9262), for which this marker was not obtained, presents a mutation at E-L335 (diagnostic of E1b1b1b1). E-M81 is the most common paternal lineage in northern Africa (with a mean frequency of about 40% in North Africa) [28-29] (S4 Fig). E-M81 is particularly represented among the Berber groups (with frequencies up to 70%) [30-31] leading to suggest an association between this paternal lineage and North African Berber communities [32]. Outside North Africa, this lineage has been observed in the Near East and Southern Europe [32]. More particularly, the significant presence of this haplogroup in extant populations of Iberia, Italy and Sicilia (with frequencies between 2 and 7%) has been correlated to the long-term Arab rule in these regions [33]. This lineage is very rare in the rest of Europe, particularly for France, with frequencies often not exceeding 1%.

**REFERENCES**

1. Mendisco F, Keyser C, Hollard C, Seldes V, Nielsen AE, Crubézy E, et al. Application of the iPLEX™ Gold SNP genotyping method for the analysis of Amerindian ancient DNA samples: benefits for ancient population studies. Electrophoresis. 2011; 32: 386-93.
2. Gabriel MN, Huffine EF, Ryan JH, Holland MM, Parsons TJ. Improved MtDNA sequence analysis of forensic remains using a "mini-primer set" amplification strategy. Journal of Forensic Sciences. 2001; 46(2): 247-253.
3. Anderson S, Bankier AT, Barrell BG, de Bruijn MH, Coulson AR, Drouin J, et al. Sequence and organization of the human mitochondrial genome. Nature. 1981; 290(5806): 457-65.
4. Andrews RM, Kubacka I, Chinnery PF, Lightowlers RN, Turnbull DM, Howell N. Reanalysis and revision of the Cambridge reference sequence for human mitochondrial DNA. Nature genetics. 1999; 23: 147-147.
5. International Society of Genetic Genealogy. Y-DNA Haplogroup Tree 2014, Version: [9.08], Date: [23 january 2014], <http://www.isogg.org/tree/>. 2014.
6. Schmieder R, Edwards R. Quality control and preprocessing of metagenomic datasets. Bioinformatics. 2011; 27: 863-864.
7. Crusoe MR, Alameldin HF, Awad S, Boucher E, Caldwell A, Cartwright R et al. The khmer software package: enabling efficient nucleotide sequence analysis. F1000Res. 2015; 4: 900.
8. García-Alcalde F, Okonechnikov K, Carbonell J, Cruz LM, Götz S, Tarazona S, et al. Qualimap: evaluating next-generation sequencing alignment data. Bioinformatics. 2012; 28(20): 2678-2679.
9. Jónsson H, Ginolhac A, Schubert M, Johnson P, Orlando L. MapDamage2.0: fast approximate Bayesian estimates of ancient DNA damage parameters. Bioinformatics. 2013; 29(13): 1682-4.
10. Li H, Handsaker B, Wysoker A, Fennell T, Ruan J, Homer N, et al. The Sequence alignment/map (SAM) format and SAMtools. Bioinformatics. 2009; 25: 2078-9.
11. Pääbo S, Stenzel U, Johnson PLF, Green RE, Kelso J, Prüfer K, et al Genetic analyses from ancient DNA. Annu Rev Genet. 2004; 38: 645-79.
12. Briggs AW, Stenzel U, Johnson PL, Green RE, Kelso J, Prüfer K, et al. Patterns of damage in genomic DNA sequences from a Neandertal. Proc Natl Acad Sci USA. 2007; 104(37): 14616–14621.
13. Fu Q, [Meyer M](http://www.ncbi.nlm.nih.gov/pubmed/?term=Meyer%20M%5BAuthor%5D&cauthor=true&cauthor_uid=23341637), [Gao X](http://www.ncbi.nlm.nih.gov/pubmed/?term=Gao%20X%5BAuthor%5D&cauthor=true&cauthor_uid=23341637), [Stenzel U](http://www.ncbi.nlm.nih.gov/pubmed/?term=Stenzel%20U%5BAuthor%5D&cauthor=true&cauthor_uid=23341637), [Burbano HA](http://www.ncbi.nlm.nih.gov/pubmed/?term=Burbano%20HA%5BAuthor%5D&cauthor=true&cauthor_uid=23341637), [Kelso J](http://www.ncbi.nlm.nih.gov/pubmed/?term=Kelso%20J%5BAuthor%5D&cauthor=true&cauthor_uid=23341637), [Pääbo S](http://www.ncbi.nlm.nih.gov/pubmed/?term=P%C3%A4%C3%A4bo%20S%5BAuthor%5D&cauthor=true&cauthor_uid=23341637). DNA analysis of an early modern human from Tianyuan Cave, China. Proc Natl Acad Sci USA. 2013; 110(6): 2223–2227.
14. Krause J, Fu Q, Good JM, Viola B, Shunkov MV, Derevianko AP, Pääbo S. The complete mitochondrial DNA genome of an unknown hominin from southern Siberia. Nature. 2010; 464 (7290): 894–897.
15. Edgar RC. MUSCLE: Multiple Sequence Alignment with High Accuracy and High Throughput. Nucleic Acids Research. 2004; 32(5): 1792–1797.
16. Krause J, Briggs AW, Kircher M, Maricic T, Zwyns N, Derevianko A, et al. A complete mtDNA genome of an early modern human from Kostenki, Russia. Curr Biol. 2010; 20(3): 231–236.
17. Sawyer S, Krause J, Guschanski K, Savolainen V, Pääbo S. Temporal patterns of nucleotide misincorporations and DNA fragmentation in ancient DNA. PLoS ONE. 2012; 7(3): e34131.
18. Batini C, Coia V, Battaggia C, Rocha J, Pilkington MM, Spedini G, et al. Phylogeography of the human mitochondrial L1c haplogroup: genetic signatures of the prehistory of Central Africa. Mol Phylogenet Evol. 2007; 43(2): 635-44.
19. Kujanová M, Pereira L, Fernandes V, Pereira JB, Cerný V. Near eastern neolithic genetic input in a small oasis of the Egyptian Western Desert. Am J Phys Anthropol. 2009; 140(2): 336-46.
20. Behar DM, Villems R, Soodyall H, Blue-Smith J, Pereira L, Metspalu E, et al. The dawn of human matrilineal diversity. Am J Hum Genet. 2008; 82(5): 1130-40.
21. González AM, Brehm A, Pérez JA, Maca-Meyer N, Flores C, Cabrera VM. Mitochondrial DNA affinities at the Atlantic fringe of Europe. Am J Phys Anthropol. 2003; 120(4): 391-404.
22. Costa MD, Pereira JB, Pala M, Fernandes V, Olivieri A, Achilli A, et al. A substantial prehistoric European ancestry amongst Ashkenazi maternal lineages. Nat Commun. 2013; 4: 2543.
23. Aboukhalid R, Sturk-Andreaggi K, Bouabdellah M, Squalli D, Irwin JA, Amzazi S. Mitochondrial DNA control region variation from samples of the Moroccan population. Int J Legal Med. 2013; 127(4): 757-9.
24. Elmadawy MA, Nagai A, Gomaa GM, Hegazy HM, Shaaban FE, Bunai Y. Investigation of mtDNA control region sequences in an Egyptian population sample. Leg Med. 2013; 15(6): 338-41.
25. Saunier JL, Irwin JA, Strouss KM, Ragab H, Sturk KA, Parsons TJ. Mitochondrial control region sequences from an Egyptian population sample. Forensic Sci Int Genet. 2009; 3(3): e97-103.
26. Ottoni C, Primativo G, Hooshiar Kashani B, Achilli A, Martínez-Labarga C, Biondi G, et al. Mitochondrial haplogroup H1 in north Africa: an early holocene arrival from Iberia. PLoS One. 2010; 5(10): e13378.
27. Achilli A, Rengo C, Magri C, Battaglia V, Olivieri A, Scozzari R, et al. The molecular dissection of mtDNA haplogroup H confirms that the Franco-Cantabrian glacial refuge was a major source for the European gene pool. Am J Hum Genet. 2004; 75: 910-918.
28. Cruciani F, La Fratta R, Santolamazza P, Sellitto D, Pascone R, Moral P, et al. Phylogeographic analysis of haplogroup E3b (E-M215) y chromosomes reveals multiple migratory events within and out of Africa. Am J Hum Genet. 2004; 74(5): 1014-22.
29. Arredi B, Poloni ES, Paracchini S, Zerjal T, Fathallah DM, Makrelouf M, et al. A predominantly neolithic origin for Y-chromosomal DNA variation in North Africa. Am J Hum Genet. 2004; 75(2): 338-45.
30. Bosch E, Calafell F, Comas D, Oefner PJ, Underhill PA, Bertranpetit J. High-resolution analysis of human Y-chromosome variation shows a sharp discontinuity and limited gene flow between northwestern Africa and the Iberian Peninsula. Am J Hum Genet. 2001; 68(4): 1019-29.
31. Scozzari R, Cruciani F, Pangrazio A, Santolamazza P, Vona G, Moral P, et al. Human Y-chromosome variation in the western Mediterranean area: implications for the peopling of the region. Hum Immunol. 2001; 62(9): 871-84.
32. Alvarez L, Santos C, Montiel R, Caeiro B, Baali A, Dugoujona JM, et al. Y-chromosome variation in South Iberia: insights into the North African contribution. Am J Hum Biol. 2009; 21(3): 407-9.
33. Capelli C, Onofri V, Brisighelli F, Boschi I, Scarnicci F, Masullo M, et al. Moors and Saracens in Europe: estimating the medieval North African male legacy in southern Europe. Eur J Hum Genet. 2009; 17(6):848-52.
