# Supplementary material for: Early Medieval Muslim Graves in France: First Archaeological, Anthropological and Palaeogenomic Evidence
Source: PLoS One. 2016 Feb 24;11(2):e0148583. doi: 10.1371/journal.pone.0148583 (PMC4765927; doi:10.1371/journal.pone.0148583)
Supplement: S1 Table — (PDF) [file pone.0148583.s009.pdf]

Table S1. **PCR and SBE primers used SNPs typing (iPLEX technology, Sequenom)**

SNP	Second PCR primer	First PCR primer	Amplicon length (bp)	SBE unextended primer (UEP)	UEP direction
**Mt SNPs**					
A_4248	acgttggatgACCACTCACCCTAGCATTAC	acgttggatgCAGACATATTTCTTAGGTTTG	108	TACCCATTACAATCTCCAGCAT	F
B_8280	acgttggatgGTATTTACCCTATAGCACCC	acgttggatgCTGTAAAGAGGTGTTGGTTC	119	ACCCTATAGCACCCCCTCTA	F
C_13263	acgttggatgATCGTAGCCTTCTCCACTTC	acgttggatgAGGAATGCTAGGTGTGGTTG	100	cGCCTTCTCCACTTCAAGTCA	F
D_5178	acgttggatgTTAAACTCCAGCACCACGAC	acgttggatgGGTGGATGGAATTAAGGGTG	101	TACTACTATCTCGCACCTGAAACAAG	F
H_2706	acgttggatgCATAGGGTCTTCTCGTCTTG	acgttggatgAGGGTTCAGCTGTCTCTTAC	109	GTCTTCTCGTCTTGCTGTGT	R
H1_3010	acgttggatgCGAACCTTTAATAGCGGCTG	acgttggatgTAGGGTTTACGACCTCGATG	84	TTTAATAGCGGCTGCACCAT	R
H3_6776	acgttggatgGCTTCCTAGGGTTTATCGTG	acgttggatgGGAGGTGAAATATGCTCGTG	100	TTATCGTGTGAGCACACCA	F
HV_14766	acgttggatgACAAGAACACCAATGACCCC	acgttggatgGGGAGGTCGATGAATGAGTG	97	ACCCCAATACGCAAAA	F
I_10034	acgttggatgAATAGTACCGTTAACTTCC	acgttggatgGTAAGGCTAGGAGGGTGTTG	120	TACCGTTAACTTCCAATTAACTAG	F
J_12612	acgttggatgGCTCTCCCTAAGCTTCAAAC	acgttggatgCTATGATGGACCATGTAACG	100	cTCTCCATAATATTCATCCCTGT	F
K_10550	acgttggatgGGAATACTAGTATATCGCTC	acgttggatgTTGAGGGTTATGAGAGTAGC	118	gggcTCGCTCACACCTCAT	F
L2_2885	acgttggatgAGACTTCACCAGTCAAAGCG	acgttggatgTCCCTAGGGTAACTTGTTCC	97	CTTCACCAGTCAAAGCGAACTAC	F
L3_3594	acgttggatgTTCTACTATGAACCCCCCTC	acgttggatgAGTAAACGGCTAGGCTAGAG	116	CCCCCTCCCCATACCCAACCCCCTGGT	F
M_10400	acgttggatgTCTGGCCTATGAGTGACTAC	acgttggatgGAGTCGAAATCATTCGTTTTG	101	ACAAAAAGGATTAGACTGAAC	F
N_10873	acgttggatgGGGAACAGCTAAATAGGTTG	acgttggatgATCAACACAACCACCCACAG	108	TGTTGATTTGGTTAAAAAATAGTAG	R
N1_10238	acgttggatgGGTAAAAGGAGGGCAATTTC	acgttggatgCGCGTCCCTTTCTCCATAAA	100	TTCTAGATCAAATAATAAGAAGGT	R
N1a_13780	acgttggatgAACAACATTTCCCCCGCATC	acgttggatgAGTCCTAGGAAAGTGACAGC	106	CCCCTTCCAAACAACA	F
R_12705	acgttggatgCAGACCCAAACATTAATCAG	acgttggatgGGTTGTTAGCGGTAACTAAG	101	AACTAAGATTAGTATGGTAATTAGGAA	R
R0_11719	acgttggatgGAGTGCGTTCGTAGTTTGAG	acgttggatgCGCAGTCATTCTCATAATCG	100	GGCAGAATAGTAATGAGGATGTAAG	R
R9_13928	acgttggatgGGTTTTGGCTCGTAAGAAGG	acgttggatgTCTCCAACATACTCGGATTC	98	GTGCGGTGTGTGATG	R
T_1888	acgttggatgTAGGTAGCTCGTCTGGTTTC	acgttggatgCTAACCCCTATACCTTCTGC	113	GGGGGTCTTAGCTTTGG	R
U_11467	acgttggatgGGTTGAGAATGAGTGTGAGG	acgttggatgCGCTGGGTCAATAGTACTTG	100	ACCATAGCCGCCTAGTTT	R
U4_11332	acgttggatgCTCACTGCCCAAGAACTATC	acgttggatgGGAGTCCGTAAAGAGGTATC	117	CAAGAACTATCAAACTCCTGAGC	F
U5_13617	acgttggatgTTACTCTCATCGCTACCTCC	acgttggatgAAGCGAGGTTGACCTGTTAG	99	GCGCCTATAGCACTCGAATAAT	F
V_4580	acgttggatgACCTGAGTAGGCCTAGAAAT	acgttggatgACTTGATGGCAGCTTCTGTG	111	tAGTAGGCCTAGAAATAAACAT	F
W_3505	acgttggatgACCAAAGAGCCCCTAAAACC	acgttggatgTAGAAGAGCGATGGTGAGAG	104	TAAAACCCGCCACATCT	F
X_6371	acgttggatgCCGTAGACCTAACCATCTTC	acgttggatgGTGATGAAATTGATGGCCCC	93	ACCTAGCAGGTGTCTC	F

Table S2. Continued

SNP	Second PCR primer	First PCR primer	Amplicon length (bp)	SBE unextended primer (UEP)	UEP direction
**Y-SNPs**					
E_L537	acgttggatgTGCTGTCTTTTTGAGAGGAG	acgttggatgACGTGAGCCATTGTAGACAG	110	TTTTGAGAGGAGATTAGGA	F
E1b1b_M215	acgttggatgATCCAGCACAGAAGCATCAG	acgttggatgCATACTTGCTGCATTAAGAC	84	GCTGGAACAGTTAGAAAG	R
F_M213	acgttggatgTATTCAGAACTTAAAACATC	acgttggatgCAAAACAACCTTTATAACAG	109	TATTCAGAACTTAAAACATCTCGTTAC	R
G_M201	acgttggatgCTCAGATCTAATAATCCAG	acgttggatgTCCAGCATCCTATCAGCTTC	100	GATCTAATAATCCAGTATCAACTGAGG	F
I_M170	acgttggatgGTGAGACACAACCCACACTG	acgttggatgCATATTCTGTGCATTATAC	102	GAGACACAACCCACACTGAAAAAAA	R
J_M304	acgttggatgCTTTCAAAACGTCTTATACC	acgttggatgTGTAACAAACAGTATGTGGG	116	TTATACCAAAATATCACCAGTTGT	R
K_M9	acgttggatgCTGCAAAGAAACGGCCTAAG	acgttggatgCATTGAACGTTTGAACATGTC	94	GGCCTAAGATGGTTGAAT	F
R_M207	acgttggatgGGGCAAATGTAAGTCAAGC	acgttggatgTCACTTCAACCTCTTGTTGG	102	gATGTAAGTCAAGCAAGAAATTTA	F
R1a_M511	acgttggatgACCATCAACATTGCCATTGC	acgttggatgCAGAGAAAAGACATTTCAGGG	103	gaagCAACATTGCCATTGCTTTAGT	F
R1b_M343	acgttggatgTCTTGCTCTTTCCCCAACAC	acgttggatgTATGCAAATGCAGAGTGCCC	111	CCCCCACATATCTCCAGGTGT	R
