# Supplementary material for: Early Medieval Muslim Graves in France: First Archaeological, Anthropological and Palaeogenomic Evidence
Source: PLoS One. 2016 Feb 24;11(2):e0148583. doi: 10.1371/journal.pone.0148583 (PMC4765927; doi:10.1371/journal.pone.0148583)
Supplement: S2 Table — (PDF) [file pone.0148583.s010.pdf]

Table S2. Radiocarbon dating

Burial	Sample	Code	Date calibrated (95.4% probability)
SP7080	A	Beta-378995	684-876 cal AD
SP7080	B	Erl-15766	579-761 cal AD
SP7089	A	Beta-378996	637-765 cal AD
SP9269	A	Beta-378997	649-767 cal AD
SP9269	B	Erl-15768	651-802 AD
