# Supplementary material for: Early Medieval Muslim Graves in France: First Archaeological, Anthropological and Palaeogenomic Evidence
Source: PLoS One. 2016 Feb 24;11(2):e0148583. doi: 10.1371/journal.pone.0148583 (PMC4765927; doi:10.1371/journal.pone.0148583)
Supplement: S3 Table — (PDF) [file pone.0148583.s011.pdf]

**Table S3. Mutated Y-SNPs detected for the three human remains analysed.**

| | Y-position (GRCh37) | Diagnostic Mutation | Allele | | | Support | | | Nb. reads | | | Nb. unique reads | | |
|---|---|---|---|---|---|---|---|---|---|---|---|---|---|---|
| | | | SP7080 | SP7089 | SP9262 | SP7080 | SP7089 | SP9262 | SP7080 | SP7089 | SP9262 | SP7080 | SP7089 | SP9262 |
| DE/M145 | 21717208 | C→T | T | - | T | 100 | - | 100 | 1 | - | 17 | 1 | - | 1 |
| E/L537 | 6861075 | G→A | A | - | A | 100 | - | 100 | 1 | - | 447 | 1 | - | 6 |
| E1b1/P179 | 14060308 | A→C | - | - | C | - | - | 100 | - | - | 6 | - | - | 2 |
| E1b1/P180 | 18601274 | G→A | - | - | A | - | - | 100 | - | - | 224 | - | - | 3 |
| E1b1b1/L336 | 21903853 | G→A | - | - | A | - | - | 100 | - | - | 16 | - | - | 1 |
| E1b1b1/M243 | 15019092 | T→C | C | C | C | 100 | 100 | 100 | 1 | 1 | 166 | 1 | 1 | 2 |
| E1b1b1b/Z827 | 7290454 | G→C | - | - | C | - | - | 100 | - | - | 756 | - | - | 2 |
| E1b1b1b1/L335 | 2710013 | A→G | - | G | G | - | 100 | 100 | - | 129 | 1 | - | 1 | 1 |
| E1b1b1b1a/M81 | 21892572 | C→T | T | T | - | 100 | 100 | | 250 | 1 | - | 2 | 1 | - |
