# Supplementary material for: Early Medieval Muslim Graves in France: First Archaeological, Anthropological and Palaeogenomic Evidence
Source: PLoS One. 2016 Feb 24;11(2):e0148583. doi: 10.1371/journal.pone.0148583 (PMC4765927; doi:10.1371/journal.pone.0148583)
Supplement: S4 Table — (PDF) [file pone.0148583.s012.pdf]

Table S4. Consensus HVR-1 sequences and SNP retrieved for the three human samples

	nps	HVR1	mt-SNP	mt-Hg	Y-SNP	Y-Hg
SP7080	16008 - 16310	16129A 16215G 16223T 16278T 16294T	2758C	L1c	M215G	E1b1b
SP7089	16008 - 16310	16224C 16256T	11467G - 10550G	K	-	-
SP9262	16009 - 16400	CRS	2706A - 3010A	H1	L537A - M215G	E1b1b
