# Supplementary material for: Early Medieval Muslim Graves in France: First Archaeological, Anthropological and Palaeogenomic Evidence
Source: PLoS One. 2016 Feb 24;11(2):e0148583. doi: 10.1371/journal.pone.0148583 (PMC4765927; doi:10.1371/journal.pone.0148583)
Supplement: S5 Table — (PDF) [file pone.0148583.s013.pdf]

Table S5. **HVR-1 sequences, mitochondrial and Y chromosome SNPs of the researchers involved in this study.**

	HVR-1	Mt-SNPs	Mt Hg	Y-SNPs	Y Hg
Manipulator 1	16209C	7028C	H	-	-
Manipulator 2	16189C, 16234T	7028C	H	-	-
Manipulator 3	16093C, 16189C, 16270T, 16274A	-	U5	-	-
Manipulator 4	CRS	2706A	H	M343	R1b
