# Supplementary material for: Early Medieval Muslim Graves in France: First Archaeological, Anthropological and Palaeogenomic Evidence
Source: PLoS One. 2016 Feb 24;11(2):e0148583. doi: 10.1371/journal.pone.0148583 (PMC4765927; doi:10.1371/journal.pone.0148583)
Supplement: S6 Table — (PDF) [file pone.0148583.s014.pdf]

Table S6. Details of modern-day populations used for comparison.

Africa	Mt	Y
Algeria	1, 2	1, 3
Angola	4, 5	4
Angola, Cabinda	6	6
Benin	7	7, 8
Burkina Faso	9	9, 10
Cameroon	11, 12	8, 13
Central African Republic	11	-
Chad	14, 15	-
Chad Lake	16	-
Egypt Berbers	17	-
Egypt	18, 19, 20, 21	1, 8, 19
Ethiopia	22, 23, 24, 25	10, 26
Gabon	11	13, 27
Ghana	28, 29	28
Guinea Bissau	30, 31	32
Ivory Coast	7	7
Kenya	25, 33, 34	8, 10
Libya	35, 36	1
Mali	37	38
Mauritania	37, 39	-
Morocco	39, 40, 41, 42, 43, 44	1, 45, 46
Morocco Berbers	17	47
Mozambique	14, 48	-
Namibia	49	-
Nigeria	15, 28	13
Sahel	50, 51	51, 52
Senegal	2, 53	26
Sierra Leone	54	-
Somalia	24, 55	56
South Africa	57	-
Sudan	24	38
Tunisia	2, 42, 58	1, 59
Western Sahara	39	46
zambia	60	10
zimbabwe	61	-

Middle East	Mt	Y
Arabian Peninsula	2, 62, 63	-
Yemen	22, 23, 64, 65	66
Druze	2, 67	-
Dubai	68	66
Iraq	2, 69, 70	56
Jordania	62, 71, 72	73
Saudi Arabia	63	-
Turkish	62, 74, 75	56, 76, 77
Iran	78, 79	-
Syria	62, 71	-
Oman	65	8
Lebanon	62, 71	76, 80
Palestinia	71	-

Europe	Mt	Y
Albania	2	81, 82
Austria	83, 84	-
Balkans	85	-
Bielorussia	147	-
Bosnia	86	82
Bulgaria	87	88
Croatia	2	82
Cyprus	89	-
Czech	2, 90	-
Estonia	85	-
France	85, 91, 92	93, 94, 95, 96
Southern France	2	97
Basques (France)	2, 98	76
Germany	84, 99, 100, 101	102
Greece (Aegean islands)	2	103
Greece	2, 89	103, 104
Hungaria	2, 105	106
Center Italy	2, 107, 108	109, 110
Northern Italy	2, 108	103, 111
Southern Italy	2, 112	76, 113
Lithuania	114	-
Macedonia	2, 115, 116	82
Netherlands	2	-
Poland	2, 117, 118	102
Portugal	119, 120	121, 122, 123
Romania	84, 116, 124	-
Sicily	2, 125	76, 94, 126
Sardinia	2	94, 127
Slovakia	85, 128, 129	102
Slovenia	86	-
Spain	2, 130	122, 123, 131
Southern Spain (andalusia)	2	45, 46, 76, 122, 123
Northern Spain	132	-
Basques (Spain)	2, 133	46, 76, 123, 131
Galicia	134	123, 135
Ukraine	2	136

Caucasus	Mt	Y
Adygei	137	-
Armenia	138	139
Azerbaidjan	138, 140	141
Dagestan	142	143
Georgia	140, 144, 145	

1. Bekada A, Fregel R, Cabrera VM, Larruga JM, Pestano J, Benhamamouch S, et al. Introducing the Algerian mitochondrial DNA and Y-chromosome profiles into the North African landscape. PLoS One. 2013; 8(2): e56775
2. Achilli A, Rengo C, Magri C, Battaglia V, Olivieri A, Scozzari R, et al. The molecular dissection of mtDNA haplogroup H confirms that the Franco-Cantabrian glacial refuge was a major source for the European gene pool. Am J Hum Genet. 2004; 75: 910-918.
3. Robino C, Crobu F, Di Gaetano C, Bekada A, Benhamamouch S, Cerutti N, et al. Analysis of Y-chromosomal SNP haplogroups and STR haplotypes in an Algerian population sample. Int J Legal Med. 2008; 122(3): 251-5.
4. Coelho M, Sequeira F, Luiselli D, Beleza S, Rocha J. On the edge of Bantu expansions: mtDNA, Y chromosome and lactase persistence genetic variation in southwestern Angola. BMC Evol Biol 2009; 9: 80.
5. Fendt L, Huber G, Röck AW, Zimmermann B, Bodner M, Delport R, et al. Mitochondrial DNA control region data from indigenous Angolan Khoe-San lineages. Forensic Sci Int Genet. 2012; 6(5): 662-3.
6. Beleza S, Gusmão L, Amorim A, Carracedo A, Salas A. The genetic legacy of western Bantu migrations. Hum Genet. 2005; 117(4): 366-75.
7. Brucato N, Cassar O, Tonasso L, Tortevoye P, Migot-Nabias F, Plancoulaine S, et al. The imprint of the Slave Trade in an African American population: mitochondrial DNA, Y chromosome and HTLV-1 analysis in the Noir Marron of French. BMC Evol Biol. 2010; 10: 314.
8. Luis JR, Rowold DJ, Regueiro M, Caeiro B, Cinnioğlu C, Roseman C, et al. The Levant versus the Horn of Africa: evidence for bidirectional corridors of human migrations. Am J Hum Genet. 2004; 74(3): 532-44.
9. Barbieri C, Whitten M, Beyer K, Schreiber H, Li M, Pakendorf B. Contrasting maternal and paternal histories in the linguistic context of Burkina Faso. Mol Biol Evol. 2012; 29(4): 1213-23.
10. de Filippo C, Barbieri C, Whitten M, Mpoloka SW, Drofn Gunnarsdóttir E, Bostoen K, et al. Y-chromosomal variation in sub-Saharan Africa: insights into the history of Niger-Congo groups. Mol Biol Evol. 2011; 28(3): 1255-69.
11. Quintana-Murci L, Quach H, Harmant C, Luca F, Massonnet B, Patin E, et al. Maternal traces of deep common ancestry and asymmetric gene flowbetween Pygmy hunter-gatherers and Bantu-speaking farmers. Proc Natl Acad Sci USA. 2008; 105(5): 1596-601.
12. Cerny V, Hajek M, Cmejla R, Bruzek J, Brdicka R. mtDNA sequences of Chadic-speaking populations from northern Cameroon suggest their affinities with eastern Africa. Ann Hum Biol. 2004; 31: 554-569.
13. Montano V, Ferri G, Marcari V, Batini C, Anyaele O, Destro-Bisol G, et al. The Bantu expansion revisited: a new analysis of Y chromosome variation in Central Western Africa. Mol Ecol. 2011; 20(13): 2693-708.
14. Cerezo M, Cerný V, Carracedo A, Salas A. Applications of MALDI-TOF MS to large-scale human mtDNA population-based studies. Electrophoresis. 2009; 30(21): 3665-73.
15. Cerny V, Salas A, Hajek M, Zaloudkova M, Brdlcka R. A bidirectional corridor in the Sahel-Sudan belt and the distinctive features of the Chad basin populations: a history revealed by the mitochondrial DNA genome. Ann Hum Genet. 2007; 71: 433-452.
16. Cerezo M, Černý V, Carracedo Á, Salas A. New insights into the Lake Chad Basin population structure revealed by high-throughput genotyping of mitochondrial DNAcoding SNPs. PLoS One. 2011; 6(4): e18682.
17. Coudray C, Olivieri A, Achilli A, Pala M, Melhaoui M, Cherkaoui M, et al. The complex and diversified mitochondrial gene pool of Berber populations. Ann Hum Genet. 2009; 73(2): 196-214.

18. Stevanovitch A, Gilles A, Bouzaid E, Kefi R, Paris F, Gayraud RP, et al. Mitochondrial DNA sequence diversity in a sedentary population fromEgypt. Ann Hum Genet. 2004; 68(1): 23-39.
19. Kujanová M, Pereira L, Fernandes V, Pereira JB, Cerný V. Near eastern neolithic genetic input in a small oasis of the Egyptian Western Desert. Am J Phys Anthropol. 2009; 140(2): 336-46.
20. Saunier JL, Irwin JA, Strouss KM, Ragab H, Sturk KA, Parsons TJ.Mitochondrial control region sequences from an Egyptian population sample. Forensic Sci Int Genet. 2009; 3(3): e97-103.
21. Elmadawy MA, Nagai A, Gomaa GM, Hegazy HM, Shaaban FE, Bunai Y. Investigation of mtDNA control region sequences in an Egyptian population sample. Leg Med. 2013; 15(6): 338-41.
22. Kivisild T, Reidla M, Metspalu E, Rosa A, Brehm A, Pennarun E, et al. Ethiopian mitochondrial DNA heritage: tracking gene flow across and around the gate of tears. Am J Hum Genet. 2004; 75(5): 752-70.
23. Non AL, Al-Meeri A, Raaum RL, Sanchez LF, Mulligan CJ. Mitochondrial DNA reveals distinct evolutionary histories for Jewish populations in Yemen and Ethiopia. Am J Phys Anthropol. 2011; 144(1): 1-10.
24. Soares P, Alshamali F, Pereira JB, Fernandes V, Silva NM, Afonso C, et al. The Expansion of mtDNA Haplogroup L3 within and out of Africa. Mol Biol Evol. 2012; 29(3): 915-27.
25. Boattini A, Castrì L, Sarno S, Useli A, Cioffi M, Sazzini M, et al. mtDNA variation in East Africa unravels the history of Afro-Asiatic groups. Am J Phys Anthropol. 2013; 150(3): 375-85.
26. Semino O, Santachiara-Benerecetti AS, Falaschi F, Cavalli-Sforza LL, Underhill PA. Ethiopians and Khoisan share the deepest clades of the human Y-chromosome phylogeny. Am J Hum Genet. 2002; 70(1): 265-8
27. Berniell-Lee G, Calafell F, Bosch E, Heyer E, Sica L, Mouguiama-Daouda P, et al. Genetic and demographic implications of the Bantu expansion: insights from human paternal lineages. Mol Biol Evol. 2009; 26(7): 1581-9.
28. Veeramah KR, Connell BA, Ansari Pour N, Powell A, Plaster CA, Zeitlyn D, et al. Little genetic differentiation as assessed by uniparental markers in the presence of substantial language variation in peoples of the Cross River region of Nigeria. BMC Evol Biol. 2010; 10: 92.
29. Fendt L, Röck A, Zimmermann B, Bodner M, Thye T, Tschentscher F, et al. MtDNA diversity of Ghana: a forensic and phylogeographic view. Forensic Sci Int Genet. 2012; 6(2): 244-9.
30. Rosa A, Brehm A, Kivisild T, Metspalu E, Villems R. MtDNA profile of West Africa Guineans: towards a better understanding of the Senegambia region. Ann Hum Genet. 2004; 68(4): 340-352.
31. Carvalho M, Brito P, Bento AM, Gomes V, Antunes H, Costa HA, et al. Paternal and maternal lineages in Guinea-Bissau population. Forensic Sci Int Genet. 2011; 5(2): 114-6.
32. Gonçalves R, Rosa A, Freitas A, Fernandes A, Kivisild T, Villems R, et al. Y-chromosome lineages in Cabo Verde Islands witness the diverse geographic origin of its first male settlers. Hum Genet. 2003; 113(6): 467-72.
33. Brandstätter A, Peterson CT, Irwin JA, Mpoke S, Koech DK, Parson W, et al. Mitochondrial DNA control region sequences from Nairobi (Kenya): inferring phylogenetic parameters for the establishment of a forensic database. Int J Legal Med. 2004; 118(5): 294-306.
34. Batai K, Babrowski KB, Arroyo JP, Kusimba CM, Williams SR. Mitochondrial DNA diversity in two ethnic groups in southeastern Kenya: perspectives from the northeastern periphery of the Bantu expansion. Am J Phys Anthropol. 2013; 150(3): 482-91.
35. Ottoni C, Martínez-Labarga C, Loogväli EL, Pennarun E, Achilli A, De Angelis F, et al. First genetic insight into Libyan Tuaregs: a maternal perspective. Ann Hum Genet. 2009; 73(4): 438-48.
36. Fadhlaoui-Zid K, Rodríguez-Botigué L, Naoui N, Benammar-Elgaaied A, Calafell F, Comas D. Mitochondrial DNA structure in North Africa reveals a genetic discontinuity in the Nile Valley. Am J Phys Anthropol. 2011; 145(1): 107-17.

37. González AM, Cabrera VM, Larruga JM, Tounkara A, Noumsi G, Thomas BN, et al. Mitochondrial DNA variation in Mauritania and Mali and their genetic relationship to other Western Africa populations. Ann Hum Genet. 2006; 70(5): 631-57.
38. Underhill PA, Shen P, Lin AA, Jin L, Passarino G, Yang WH,et al. Y chromosome sequence variation and the history of human populations. Nat Genet. 2000; 26(3): 358-61.
39. Ennafaa H, Cabrera VM, Abu-Amero KK, González AM, Amor MB, Bouhaha R, et al. Mitochondrial DNA haplogroup H structure in North Africa. BMC Genet. 2009; 10: 8.
40. Brakez Z, Bosch E, Izaabel H, Akhayat O, Comas D, Bertranpetit J, et al. Human mitochondrial DNA sequence variation in the Moroccan population of the Souss area. Ann Hum Biol. 2001; 28(3): 295-307.
41. Rhouda T, Martínez-Redondo D, Gómez-Durán A, Elmtili N, Idaomar M, Díez-Sánchez C, et al. Moroccan mitochondrial genetic background suggests prehistoric human migrations across the Gibraltar Strait. Mitochondrion. 2009; 9(6): 402-7.
42. Turchi C, Buscemi L, Giacchino E, Onofri V, Fendt L, Parson W, et al. Polymorphisms of mtDNA control region in Tunisian and Moroccan populations: an enrichment of forensic mtDNA databases with Northern Africa data. Forensic Sci Int Genet. 2009; 3(3): 166-72.
43. Harich N, Costa MD, Fernandes V, Kandil M, Pereira JB, Silva NM, et al. The trans-Saharan slave trade - clues from interpolation analyses and high-resolution characterization of mitochondrial DNA lineages. BMC Evol Biol. 2010; 10: 138.
44. Aboukhalid R, Sturk-Andreaggi K, Bouabdellah M, Squalli D, Irwin JA, Amzazi S. Mitochondrial DNA control region variation from samples of the Moroccan population. Int J Legal Med. 2013; 127(4): 757-9.
45. Alvarez L, Santos C, Montiel R, Caeiro B, Baali A, Dugoujona JM, et al. Y-chromosome variation in South Iberia: insights into the North African contribution. Am J Hum Biol. 2009; 21(3): 407-9.
46. Bosch E, Calafell F, Comas D, Oefner PJ, Underhill PA, Bertranpetit J. High-resolution analysis of human Y-chromosome variation shows a sharp discontinuity and limited gene flow between northwestern Africa and the Iberian Peninsula. Am J Hum Genet. 2001; 68(4): 1019-29
47. Scozzari R, Cruciani F, Pangrazio A, Santolamazza P, Vona G, Moral P, et al. Human Y-chromosome variation in the western Mediterranean area: implications for the peopling of the region. Hum Immunol. 2001; 62(9): 871-84.
48. Salas A, Richards M, De la Fe T, Lareu MV, Sobrino B, Sánchez-Diz P, et al. The making of the African mtDNA landscape. Am J Hum Genet. 2002; 71(5): 1082-111.
49. Barbieri C, Güldemann T, Naumann C, Gerlach L, Berthold F, Nakagawa H, et al. Unraveling the complex maternal history of Southern African Khoisan populations. Am J Phys Anthropol. 2014; 153(3): 435-48.
50. Černý V, Pereira L, Musilová E, Kujanová M, Vašíková A, Blasi P, et al. Genetic structure of pastoral and farmer populations in the African Sahel. Mol Biol Evol. 2011; 28(9): 2491-500.
51. Pereira L, Černý V, Cerezo M, Silva NM, Hájek M, Vašíková A, et al. Linking the sub-Saharan and West Eurasian gene pools: maternal and paternal heritage of the Tuareg nomads from the African Sahel. Eur J Hum Genet. 2010; 18(8): 915-23.
52. Bučková J, Cerný V, Novelletto A. Multiple and differentiated contributions to the male gene pool of pastoral and farmer populations of the African Sahel. Am J Phys Anthropol. 2013; 151(1): 10-21.
53. Stefflova K, Dulik MC, Pai AA, Walker AH, Zeigler-Johnson CM, Gueye SM, et al. Evaluation of group genetic ancestry of populations from Philadelphia and Dakar in the context of sex-biased admixture in the Americas. PLoS One. 2009; 4(11): e7842.

54. Jackson BA, Wilson JL, Kirbah S, Sidney SS, Rosenberger J, Bassie L, et al. Mitochondrial DNA genetic diversity among four ethnic groups in SierraLeone. Am J Phys Anthropol. 2005; 128(1): 156-63.
55. Mikkelsen M, Fendt L, Röck AW, Zimmermann B, Rockenbauer E, Hansen AJ, et al. Forensic and phylogeographic characterisation of mtDNA lineages from Somalia. Int J Legal Med. 2012; 126(4): 573-9.
56. Sanchez JJ, Hallenberg C, Børsting C, Hernandez A, Morling N. High frequencies of Y chromosome lineages characterized by E3b1, DYS19-11, DYS392-12 in Somali males. Eur J Hum Genet. 2005; 13(7): 856-66.
57. Quintana-Murci L, Harmant C, Quach H, Balanovsky O, Zaporozhchenko V, Bormans C, et al. Strong maternal Khoisan contribution to the South African coloured population: a case of gender-biased admixture. Am J Hum Genet. 2010; 86(4): 611-20.
58. Cherni L, Fernandes V, Pereira JB, Costa MD, Goios A, Frigi S, et al. Post-last glacial maximum expansion from Iberia to North Africa revealed by fine characterization of mtDNA H haplogroup inTunisia. Am J Phys Anthropol. 2009; 139(2): 253-60.
59. Fadhlaoui-Zid K, Martinez-Cruz B, Khodjet-el-khil H, Mendizabal I, Benammar-Elgaaied A, Comas D. Genetic structure of Tunisian ethnic groups revealed by paternal lineages. Am J Phys Anthropol. 2011; 146(2): 271-80.
60. de Filippo C, Heyn P, Barham L, Stoneking M, Pakendorf B. Genetic perspectives on forager-farmer interaction in the Luangwa valley ofZambia. Am J Phys Anthropol. 2010; 141(3): 382-94.
61. Castrì L, Tofanelli S, Garagnani P, Bini C, Fosella X, Pelotti S, et al. mtDNA variability in two Bantu-speaking populations (Shona and Hutu) from Eastern Africa: implications for peopling and migration patterns in sub-Saharan Africa. Am J Phys Anthropol. 2009; 140(2): 302-11.
62. Roostalu U, Kutuev I, Loogväli E-L, Metspalu E, Tambets K, Reidla M, et al. Origin and expansion of haplogroup H, the dominant human mitochondrial DNA lineage in West Eurasia: the Near Eastern and Caucasian perspective. Mol Biol Evol. 2007; 24: 436-448.
63. Abu-Amero KK, Larruga JM, Cabrera VM, González AM. Mitochondrial DNA structure in the Arabian Peninsula. BMC Evol Biol. 2008; 8: 45.
64. Cerný V, Mulligan CJ, Rídl J, Zaloudková M, Edens CM, Hájek M, et al. Regional differences in the distribution of the sub-Saharan, West Eurasian, and South Asian mtDNA lineages in Yemen. Am J Phys Anthropol. 2008; 136(2): 128-37.
65. Al-Abri A, Podgorná E, Rose JI, Pereira L, Mulligan CJ, Silva NM, et al. Pleistocene-Holocene boundary in Southern Arabia from the perspective of human mtDNA variation. Am J Phys Anthropol. 2012; 149(2): 291-8.
66. Cadenas AM, Zhivotovsky LA, Cavalli-Sforza LL, Underhill PA, Herrera RJ. Y-chromosome diversity characterizes the Gulf of Oman. Eur J Hum Genet. 2007; 16(3): 374-86.
67. Shlush LI, Behar DM, Yudkovsky G, Templeton A, Hadid Y, Basis F, et al. The Druze: a population genetic refugium of the Near East. PLoS One. 2008; 3(5): e2105.
68. Alshamali F, Brandstatter A, Zimmermann B, Parson W. Mitochondrial DNA control region variation in Dubai, United Arab Emirates. Forensic Sci Int Genet. 2008; 2: e9-10.
69. Al-Zahery N, Pala M, Battaglia V, Grugni V, Hamod MA, Kashani BH, et al. In search of the genetic footprints of Sumerians: a survey of Y-chromosome and mtDNA variation in the Marsh Arabs of Iraq. BMC Evol Biol. 2011; 11: 288.
70. Al-Zahery N, Saunier J, Ellingson K, Parson W, Parsons TJ, Irwin JA. Characterization of mitochondrial DNA control region lineages in Iraq. Int J Legal Med. 2013; 127(2): 373-5.
71. Badro DA, Douaihy B, Haber M, Youhanna SC, Salloum A, Ghassibe-Sabbagh M, et al. Y-chromosome and mtDNA genetics reveal significant contrasts in affinities of modern Middle Eastern populations with European and African populations. PLoS One. 2013; 8(1): e54616.

72. González AM, Karadsheh N, Maca-Meyer N, Flores C, Cabrera VM, Larruga JM. Mitochondrial DNA variation in Jordanians and their genetic relationship to other Middle East populations. Ann Hum Biol. 2008; 35(2): 212-31.
73. Flores C, Maca-Meyer N , Larruga JM, Cabrera VM, Karadsheh N, Gonzalez AM. Isolates in a corridor of migrations: a high-resolution analysis of Y-chromosome variation in Jordan. J Hum Genet. 2005; 50(9): 435-41.
74. Mergen H, Oner R, Oner C. Mitochondrial DNA sequence variation in the Anatolian Peninsula (Turkey). J Genet. 2004; 83(1): 39-47.
75. Ottoni C, Ricaut FX, Vanderheyden N, Brucato N, Waelkens M, Decorte R. Mitochondrial analysis of a Byzantine population reveals the differential impact of multiple historical events in South Anatolia. Eur J Hum Genet. 2011; 19(5): 571-6.
76. Cruciani F, La Fratta R, Santolamazza P, Sellitto D, Pascone R, Moral P, et al. Phylogeographic analysis of haplogroup E3b (E-M215) y chromosomes reveals multiple migratory events within and out of Africa. Am J Hum Genet. 2004; 74(5): 1014-22.
77. Cinnioğlu C, King R, Kivisild T, Kalfoğlu E, Atasoy S, Cavalleri GL, et al. Excavating Y-chromosome haplotype strata in Anatolia. Hum Genet. 2004; 114(2): 127-48.
78. Metspalu M, Kivisild T, Metspalu E, Parik J, Hudjashov G, Kaldma K, et al. Most of the extant mtDNA boundaries in south and southwest Asia were likely shaped during the initial settlement of Eurasia by anatomically modern humans. BMC Genet. 2004; 5: 26.
79. Farjadian S, Sazzini M, Tofanelli S, Castrì L, Taglioli L, Pettener D, et al. Discordant patterns of mtDNA and ethno-linguistic variation in 14 Iranian Ethnic groups. Hum Hered. 2011; 72(2): 73-84.
80. Zalloua PA, Xue Y, Khalife J, Makhoul N, Debiane L, Platt DE, et al. Y-chromosomal diversity in Lebanon is structured by recent historical events. Am J Hum Genet. 2008; 82(4): 873-82.
81. Ferri G, Tofanelli S, Alù M, Taglioli L, Radheshi E, Corradini B, et al. Y-STR variation in Albanian populations: implications on the match probabilities and the genetic legacy of the minority claiming an Egyptian descent. Int J Legal Med. 2010; 124(5): 363-70.
82. Peričić M, Lauc LB, Klarić IM, Rootsi S, Janićijevic B, Rudan I, et al. High-resolution phylogenetic analysis of southeastern Europe traces major episodes of paternal gene flow among Slavic populations. Mol Biol Evol. 2005; 22(10): 1964-75.
83. Brandstätter A, Niederstätter H, Pavlic M, Grubwieser P, Parson W. Generating population data for the EMPOP database - an overview of the mtDNA sequencing and data evaluation processes considering 273 Austrian control region sequences as example. Forensic Sci Int. 2006; 166(2-3): 164-75.
84. Brandstätter A, Zimmermann B, Wagner J, Göbel T, Röck AW, Salas A, et al. Timing and deciphering mitochondrial DNA macro-haplogroup R0 variability in Central Europe and Middle East. BMC Evol Biol. 2008; 8: 191.
85. Loogväli EL, Roostalu U, Malyarchuk BA, Derenko MV, Kivisild T, Metspalu E, et al. Disuniting uniformity: A pied cladistic canvas of mtDNA Haplogroup H in Eurasia. Mol Biol Evol. 2004; 21: 2012-2021.
86. Malyarchuk BA, Grzybowski T, Derenko MV, Czarny J, Drobnic K, Miścicka-Sliwka D. Mitochondrial DNA variability in Bosnians and Slovenians. Ann Hum Genet. 2003; 67(5): 412-25.
87. Karachanak S, Carossa V, Nesheva D, Olivieri A, Pala M, Hooshiar Kashani B, et al. Bulgarians vs the other European populations: a mitochondrial DNA perspective. Int J Legal Med. 2012; 126(4): 497-503.
88. Karachanak S, Grugni V, Fornarino S, Nesheva D, Al-Zahery N, Battaglia V, et al. Y-chromosome diversity in modern Bulgarians: new clues about their ancestry. PLoS One. 2013; 8(3): e56779.

89. Irwin J, Saunier J, Strouss K, Paintner C, Diegoli T, Sturk K, et al. Mitochondrial control region sequences from northern Greece and Greek Cypriots. Int J Legal Med. 2008; 122(1): 87-9.
90. Malyarchuk BA, Vanecek T, Perkova MA, Derenko MV, Sip M. Mitochondrial DNA variability in the Czech population, with application to the ethnic history of Slavs. Hum Biol. 2006; 78(6): 681-96.
91. Dubut V, Chollet L, Murail P, Cartault F, Béraud-Colomb E, Serre M, et al. mtDNA polymorphisms in five French groups: importance of regional sampling. Eur J Hum Genet. 2004; 12(4): 293-300.
92. Richard C, Pennarun E, Kivisild T, Tambets K, Tolk HV, Metspalu E, et al. An mtDNA perspective of French genetic variation. Ann Hum Biol. 2007; 34(1): 68-79.
93. Scozzari R, Cruciani F, Pangrazio A, Santolamazza P, Vona G, Moral P, et al. Human Y-chromosome variation in the western Mediterranean area: implications for the peopling of the region. Hum Immunol. 2001; 62(9): 871-84.
94. Francalacci P, Morelli L, Underhill PA, Lillie AS, Passarino G, Useli A, et al. Peopling of three Mediterranean islands (Corsica, Sardinia, and Sicily) inferred by Y-chromosome biallelic variability. Am J Phys Anthropol. 2003; 121(3): 270-9.
95. King RJ, Di Cristofaro J, Kouvatsi A, Triantaphyllidis C, Scheidel W, Myres NM, et al. The coming of the Greeks to Provence and Corsica: Y-chromosome models of archaic Greek colonization of the western Mediterranean. BMC Evol Biol. 2011; 11: 69.
96. Larmuseau MH, Vanderheyden N, Jacobs M, Coomans M, Larno L, Decorte R. Micro-geographic distribution of Y-chromosomal variation in the central-western European region Brabant. Forensic Sci Int Genet. 2011; 5(2): 95-9.
97. Francalacci P, Morelli L, Underhill PA, Lillie AS, Passarino G, Useli A, et al. Peopling of three Mediterranean islands (Corsica, Sardinia, and Sicily) inferred by Y-chromosome biallelic variability. Am J Phys Anthropol. 2003, 121(3): 270-9.
98. Alfonso-Sánchez MA, Cardoso S, Martínez-Bouzas C, Peña JA, Herrera RJ, Castro A, et al. Mitochondrial DNA haplogroup diversity in Basques: a reassessment based on HVI and HVII polymorphisms. Am J Hum Biol. 2008; 20(2): 154-64.
99. Brandstätter A, Klein R, Duftner N, Wiegand P, Parson W. Application of a quasi-median network analysis for the visualization of character conflicts to a population sample of mitochondrial DNA control region sequences from southern Germany (Ulm). Int J Legal Med. 2006; 120(5): 310-4.
100. Poetsch M, Wittig H, Krause D, Lignitz E. Mitochondrial diversity of a northeast German population sample. Forensic Sci Int. 2003; 137(2-3): 125-32.
101. Tetzlaff S, Brandstätter A, Wegener R, Parson W, Weirich V. Mitochondrial DNA population data of HVS-I and HVS-II sequences from a northeast German sample. Forensic Sci Int. 2007; 172(2-3): 218-24.
102. Rębała K, Martínez-Cruz B, Tönjes A, Kovacs P, Stumvoll M, Lindner I, et al. Contemporary paternal genetic landscape of Polish and German populations: from early medieval Slavic expansion to post-World War II resettlements. Eur J Hum Genet. 2013; 21(4): 415-22.
103. Di Giacomo F, Luca F, Anagnou N, Ciavarella G, Corbo RM, Cresta M, et al. Clinal patterns of human Y chromosomal diversity in continental Italy and Greece are dominated by drift and founder effects. Mol Phylogenet Evol. 2003; 28(3): 387-95.
104. King RJ, Ozcan SS, Carter T, Kalfoğlu E, Atasoy S, Triantaphyllidis C, et al. Differential Y-chromosome Anatolian influences on the Greek and Cretan Neolithic. Ann Hum Genet. 2008; 72: 205-14.
105. Irwin J, Egyed B, Saunier J, Szamosi G, O'Callaghan J, Padar Z, et al. Hungarian mtDNA population databases from Budapest and the Baranya county Roma. Int J Legal Med. 2007; 121(5): 377-83.

106. Völgyi A, Zalán A, Szvetnik E, Pamjav H. Hungarian population data for 11 Y-STR and 49 Y-SNP markers. Forensic Sci Int Genet. 2009; 3(2): e27-8.
107. Achilli A, Olivieri A, Pala M, Metspalu E, Fornarino S, Battaglia V, et al. Mitochondrial DNA variation of modern Tuscans supports the near eastern origin of Etruscans. Am J Hum Genet. 2007; 80(4): 759-68.
108. Turchi C, Buscemi L, Previderè C, Grignani P, Brandstätter A, Achilli A, et al. Italian mitochondrial DNA database: results of a collaborative exercise and proficiency testing. Int J Legal Med. 2008; 122(3): 199-204.
109. Capelli C, Brisighelli F, Scarnicci F, Arredi B, Caglia' A, Vetrugno G, et al. Y chromosome genetic variation in the Italian peninsula is clinal and supports an admixture model for the Mesolithic-Neolithic encounter. Mol Phylogenet Evol. 2007; 44(1): 228-39.
110. Onofri V, Alessandrini F, Turchi C, Fraternale B, Buscemi L, Pesaresi M, et al. Y-chromosome genetic structure in sub-Apennine populations of Central Italy by SNP and STR analysis. Int J Legal Med. 2007; 121(3): 234-7.
111. Ferri G, Alù M, Corradini B, Radheshi E, Beduschi G. Slow and fast evolving markers typing in Modena males (North Italy). Forensic Sci Int Genet. 2009; 3(2): e31-3.
112. Babalini C, Martínez-Labarga C, Tolk HV, Kivisild T, Giampaolo R, Tarsi T, et al. The population history of the Croatian linguistic minority of Molise (southern Italy): a maternal view. Eur J Hum Genet. 2005; 13(8): 902-12.
113. Ferri G, Ceccardi S, Lugaresi F, Bini C, Ingravallo F, Cicognani A, et al. Male haplotypes and haplogroups differences between urban (Rimini) and rural area (Valmarecchia) in Romagna region (North Italy). Forensic Sci Int. 2008; 175(2-3): 250-5.
114. Kasperaviciute D, Kucinskas V, Stoneking M. Y chromosome and mitochondrial DNA variation in Lithuanians. Ann Hum Genet. 2004; 68: 438-452.
115. Zimmermann B, Brandstätter A, Duftner N, Niederwieser D, Spiroski M, Arsov T, et al. Mitochondrial DNA control region population data from Macedonia. Forensic Sci Int Genet. 2007; 1(3-4): e4-9.
116. Bosch E, Calafell F, González-Neira A, Flaiz C, Mateu E, Scheil HG, et al. Paternal and maternal lineages in the Balkans show a homogeneous landscape over linguistic barriers, except for the isolated Aromuns. Ann Hum Genet. 2006; 70(4): 459-87.
117. Grzybowski T, Malyarchuk BA, Derenko MV, Perkova MA, Bednarek J, Woźniak M. Complex interactions of the Eastern and Western Slavic populations with other European groups as revealed by mitochondrial DNA analysis. Forensic Sci Int Genet. 2007; 1(2): 141-7.
118. Malyarchuk BA, Grzybowski T, Derenko MV, Czarny J, Miścicka-Sliwka D. Mitochondrial DNA diversity in the Polish Roma. Ann Hum Genet. 2006; 70(2): 195-206.
119. Rosa A, Fonseca BV, Krug T, Manso H, Gouveia L, Albergaria I, et al. Mitochondrial haplogroup H1 is protective for ischemic stroke in Portuguese patients. BMC Med Genet. 2008; 9: 57
120. González AM, Brehm A, Pérez JA, Maca-Meyer N, Flores C, Cabrera VM. Mitochondrial DNA affinities at the Atlantic fringe of Europe. Am J Phys Anthropol. 2003; 120(4): 391-404.
121. Beleza S, Gusmão L, Lopes A, Alves C, Gomes I, Giouzeli M, et al. Micro-phylogeographic and demographic history of Portuguese male lineages. Ann Hum Genet. 2006; 70(2): 181-94.
122. Flores C, Maca-Meyer N, González AM, Oefner PJ, Shen P, Pérez JA, et al. Reduced genetic structure of the Iberian peninsula revealed by Y-chromosome analysis: implications for population demography. Eur J Hum Genet. 2004; 12(10): 855-63
123. Adams SM, Bosch E, Balaresque PL, Ballereau SJ, Lee AC, Arroyo E, et al. The genetic legacy of religious diversity and intolerance: paternal lineages of Christians, Jews, and Muslims in the Iberian Peninsula. Am J Hum Genet. 2008; 83(6): 725-36.

124. Brandstätter A, Egyed B, Zimmermann B, Duftner N, Padar Z, Parson W. Migration rates and genetic structure of two Hungarian ethnic groups in Transylvania, Romania. Ann Hum Genet. 2007; 71(6): 791-803.
125. Cali F, Le Roux MG, D'Anna R, Flugy A, De Leo G, Chiavetta V, et al. MtDNA control region and RFLP data for Sicily and France. Int J Legal Med. 2001; 114(4-5): 229-31.
126. Di Gaetano C, Cerutti N, Crobu F, Robino C, Inturri S, Gino S, et al. Differential Greek and northern African migrations to Sicily are supported by genetic evidence from the Y chromosome. Eur J Hum Genet. 2009; 17(1): 91-9.
127. Contu D, Morelli L, Santoni F, Foster JW, Francalacci P, Cucca F. Y-chromosome based evidence for pre-neolithic origin of the genetically homogeneous but diverse Sardinian population: inference for association scans. PLoS One. 2008; 3(1): e1430.
128. Malyarchuk BA, Perkova MA, Derenko MV, Vanecek T, Lazur J, Gomolcak P. Mitochondrial DNA variability in Slovaks, with application to the Roma origin. Ann Hum Genet. 2008; 72: 228-240.
129. Lehocký I, Baldovic M, Kádasi L, Metspalu E. A database of mitochondrial DNA hypervariable regions I and II sequences of individuals from Slovakia. Forensic Sci Int Genet. 2008; 2(4): e53-9.
130. Alvarez-Iglesias V, Mosquera-Miguel A, Cerezo M, Quintáns B, Zarrabeitia MT, Cuscó I, et al. New population and phylogenetic features of the internal variation within mitochondrial DNA macro-haplogroup R0. PLoS One. 2009; 4: e5112.
131. Alonso S, Flores C, Cabrera V, Alonso A, Martín P, Albarrán C, et al. The place of the Basques in the European Y-chromosome diversity landscape. Eur J Hum Genet. 2005; 13(12): 1293–1302.
132. Alvarez L, Santos C, Ramos A, Pratdesaba R, Francalacci P, Aluja MP. Mitochondrial DNA patterns in the Iberian Northern plateau: population dynamics and substructure of the Zamora province. Am J Phys Anthropol. 2010; 142(4): 531-9.
133. Alfonso-Sánchez MA, Cardoso S, Martínez-Bouzas C, Peña JA, Herrera RJ, Castro A, et al. Mitochondrial DNA haplogroup diversity in Basques: a reassessment based on HVI and HVII polymorphisms. Am J Hum Biol. 2008; 20(2): 154-64.
134. Quintans B, Alvarez-Iglesias V, Salas A, Phillips C, Lareu MV, Carracedo A. Typing of mitochondrial DNA coding region SNPs of forensic and anthropological interest using SNaPshot minisequencing. Forensic Sci Int. 2004; 140: 251-257.
135. Lopez-Parra AM, Gusmão L, Tavares L, Baeza C, Amorim A, Mesa MS, et al. In search of the pre- and post-neolithic genetic substrates in Iberia: evidence from Y-chromosome in Pyrenean populations. Ann Hum Genet. 2009; 73(1): 42-53.
136. Mielnik-Sikorska M, Daca P, Woźniak M, Malyarchuk BA, Bednarek J, Dobosz T, et al. Genetic data from Y chromosome STR and SNP loci in Ukrainian population. Forensic Sci Int Genet. 2013; 7(1): 200-3.
137. Macaulay V, Richards M, Hickey E, Vega E, Cruciani F, Guida V, et al. The emerging tree of West Eurasian mtDNAs: A synthesis of control-region sequences and RFLPs. Am J Hum Genet. 1999; 64: 232-249.
138. Richards M, Macaulay V, Hickey E, Vega E, Sykes B, Guida V, et al. Tracing European founder lineages in the Near Eastern mtDNA pool. Am J Hum Genet. 2000; 67(5): 1251-76.
139. Hovhannisyan A, Khachatryan Z, Haber M, Hrechdakian P, Karafet T, Zalloua P, et al. Different waves and directions of Neolithic migrations in the Armenian Highland. Investig Genet. 2014; 5(1): 15.
140. Quintana-Murci L, Chaix R, Wells RS, Behar DM, Sayar H, Scozzari R, et al. Where west meets east: the complex mtDNA landscape of the southwest and Central Asian corridor. Am J Hum Genet. 2004; 74(5): 827-45.

141. Nasidze I, Quinque D, Rahmani M, Alemohamad SA, Asadova P, Zhukova O, et al. mtDNA and Y-chromosome variation in the Talysh of Iran and Azerbaijan. Am J Phys Anthropol. 2009; 138(1): 82-9.
142. Grzybowski T, Malyarchuk BA, Derenko MV, Perkova MA, Bednarek J, Woźniak M. Complex interactions of the Eastern and Western Slavic populations with other European groups as revealed by mitochondrial DNA analysis. Forensic Sci Int Genet. 2007; 1(2): 141-7.
143. Bertoncini S, Bulayeva K, Ferri G, Pagani L, Caciagli L, Taglioli L, et al. The dual origin of Tati-speakers from Dagestan as written in the genealogy of uniparental variants. Am J Hum Biol. 2012; 24(4): 391-9.
144. Comas D, Calafell F, Bendukidze N, Fañanás L, Bertranpetit J. Georgian and kurd mtDNA sequence analysis shows a lack of correlation between languages and female genetic lineages. Am J Phys Anthropol. 2000; 112(1): 5-16.
145. Alfonso-Sánchez MA, Martínez-Bouzas C, Castro A, Peña JA, Fernández-Fernández I, Herrera RJ, et al. Sequence polymorphisms of the mtDNA control region in a human isolate: the Georgians from Swanetia. J Hum Genet. 2006; 51(5): 429-39.
146. Tarkhnishvili D, Gavashelishvili A, Murtskhvaladze M, Gabelaia M, Tevzadze G. Human paternal lineages, languages, and environment in the Caucasus. Hum Biol. 2014; 86(2): 113-30.
147. Kushniarevich A, Sivitskaya L, Danilenko N, Novogrodskii T, Tsybovsky I, Kiseleva A, et al. Uniparental genetic heritage of belarusians: encounter of rare middle eastern matrilineages with a central European mitochondrial DNA pool. PLoS One. 2013; 8(6): e66499.
